# Out of site, out of mind: Changes in feather moss phyllosphere microbiota in mine offsite boreal landscapes

**DOI:** 10.3389/fmicb.2023.1148157

**Published:** 2023-04-05

**Authors:** Xiangbo Yin, Christine Martineau, Abdul Samad, Nicole J. Fenton

**Affiliations:** ^1^NSERC-UQAT Industrial Chair in Northern Biodiversity in a Mining Context, Rouyn-Noranda, QC, Canada; ^2^Centre d’Étude de la Forêt, Institut de Recherche sur les Forêts (IRF), Université du Québec en Abitibi-Témiscamingue (UQAT), Rouyn-Noranda, QC, Canada; ^3^Natural Resources Canada, Canadian Forest Service, Laurentian Forestry Centre, Quebec City, QC, Canada

**Keywords:** phyllosphere, landscape ecology, microbial indicators, bryophytes, taiga forest

## Abstract

Plant-microbe interactions play a crucial role in maintaining biodiversity and ecological services in boreal forest biomes. Mining for minerals, and especially the emission of heavy metal-enriched dust from mine sites, is a potential threat to biodiversity in offsite landscapes. Understanding the impacts of mining on surrounding phyllosphere microbiota is especially lacking. To investigate this, we characterized bacterial and fungal communities in the phyllosphere of feather moss *Pleurozium schreberi* (Brid). Mitt in boreal landscapes near six gold mine sites at different stages of the mine lifecycle. We found that (1) both mining stage and ecosystem type are drivers of the phyllosphere microbial community structure in mine offsite landscapes; (2) Bacterial alpha diversity is more sensitive than fungal alpha diversity to mining stage, while beta diversity of both groups is impacted; (3) mixed and deciduous forests have a higher alpha diversity and a distinct microbial community structure when compared to coniferous and open canopy ecosystems; (4) the strongest effects are detectable within 0.2 km from operating mines. These results confirmed the presence of offsite effects of mine sites on the phyllosphere microbiota in boreal forests, as well as identified mining stage and ecosystem type as drivers of these effects. Furthermore, the footprint was quantified at 0.2 km, providing a reference distance within which mining companies and policy makers should pay more attention during ecological assessment and for the development of mitigation strategies. Further studies are needed to assess how these offsite effects of mines affect the functioning of boreal ecosystems.

## 1. Introduction

The phyllosphere–the aerial surfaces of plants–represents a widespread and diverse habitat for various groups of microorganisms, such as bacteria and fungi (Bashir et al., 2021; Perreault and Laforest-Lapointe, 2021). Microorganisms in the phyllosphere not only promote host plant fitness and nutrient acquisition, but also play important roles in global biodiversity and biogeochemical cycles (Peñuelas and Terradas, 2014; Stone et al., 2018; Perreault and Laforest-Lapointe, 2021). In boreal forests, where nutrient availability is limited, the moss phyllosphere microbiota plays a major role in carbon and nitrogen cycling, as exemplified by the feather mosses-cyanobacteria (Rousk et al., 2013) and *Sphagnum*–methanotrophs (Putkinen et al., 2012) associations. *Pleurozium schreberi* (one of the most widespread boreal feather mosses) alone, through the activity of its phyllosphere nitrogen-fixing bacteria (e.g., Cyanobacteria), was shown to fix 1.5 to 2.0 kg nitrogen ha^–1^ yr^–1^ in boreal forests (DeLuca et al., 2002).

The global area of mines covers approximately 57,300 km^2^, which is one of the growing anthropogenic threats to biodiversity and ecosystem services in the Anthropocene (Corlett, 2015; Chester et al., 2019; Maus et al., 2020). Mine dust pollution is considered a major threat on the environment through damaging surface vegetation, landscape degradation and air pollution (Yu and Zahidi, 2022). Fugitive dust emission are generated from various mining and associated activities including ore blasting, excavation, crushing, screening, tailings pond dusting, paved/unpaved roadways and ore/waste transportations (Petavratzi et al., 2005; Yu and Zahidi, 2022). This fugitive dust consists of particulate matters which are important carriers of toxic and harmful metal elements (such as As, Cd, Cr, Cu, Ni, Pb, Zn) and other organic pollutants (such as volatile organic compounds, Zheng et al., 2018; Abbasi et al., 2021; Neitlich et al., 2022). For example, case studies of individual gold mines have found high concentration of heavy metals (e.g., As, Hg, Cd, and Cr) in mine offsite landscapes, including in soils and plant foliage (Abdul-Wahab and Marikar, 2012; Candeias et al., 2014; Olobatoke and Mathuthu, 2016; Xiao et al., 2017; Pugh et al., 2022). Many studies found that dust can transport heavy metals over distances greater than 1 km and even up to over 20 km away from the mining center (Avkopashvili et al., 2017; Yuan-Gen et al., 2019; Li Y. et al., 2020). The microbiota in the range affected by dust deposition can be affected by the high concentration of pollutants (e.g., heavy metals), leading to altered community structures (i.e., alpha and beta diversity) in soil, water, sediments and rhizosphere (Fashola et al., 2016; Mohamad et al., 2017; Liu et al., 2021; Munford et al., 2023). While the phyllosphere microbiota is highly exposed to the environment and could therefore be strongly impacted by mine dust, it is unclear whether mines affect microbial communities in the surrounding phyllosphere, particularly in boreal forests.

The amount of dust produced by mines varies throughout the mine lifecycle, from establishment to operation, closure and rehabilitation, and the mine lifecycle could therefore be an important driver of the impact of mining on the phyllosphere microbiota. Operating sites with multiple activities (e.g., drilling, blasting, digging, crushing and transportations) generally produce more dust pollutants than non-operating sites (the mines at the stage of establishment, closure and rehabilitation, Punia, 2021). Previous studies have shown that the offsite effects of mines on understory plant and bryophyte richness are more pronounced near operating mines than non-operating sites (Boisvert et al., 2021; Yin et al., 2022). We expect to find the same pattern in the feather moss phyllosphere microbiota.

Ecosystem types could be another driver of the phyllosphere community structure given their difference in environmental conditions (Barbier et al., 2008) and the capacity to store and capture dust (Han et al., 2022). On the one hand, forest type affects the community composition of feather moss phyllosphere *via* canopy structure differences that shape environmental conditions such as temperature, soil pH, leaf litter input, nitrogen, moisture, and light (Jean et al., 2020a; Holland-Moritz et al., 2021; Rodríguez-Rodríguez et al., 2022). Proteobacteria, Acidobacteriota, Bacteroidota, and Actinobacteriota are commonly dominant in the phyllosphere of feather mosses in both deciduous and coniferous forests (Cutler et al., 2017; Holland-Moritz et al., 2018, 2021; Jean et al., 2020a), but Proteobacteria, Acidobacteriota, and WPS-2 (Candidatus Eremiobacterota; Ji et al., 2021) were found at higher relative abundances in coniferous than in deciduous forests, which supported higher relative abundances of Bacterioidota and Cyanobacteria (Jean et al., 2020a; Rodríguez-Rodríguez et al., 2022). On the other hand, the differences in the temperature, humility and leaf morphology characteristics of various ecosystem types can affect dust transport (Luo et al., 2021; Han et al., 2022). Coniferous trees having a rough surface and a higher stomata density generally capture more particulate matters than deciduous trees (Lu et al., 2018). At the stand level, coniferous forests and open-canopy peatlands generally exhibit higher air humidity compared to deciduous and mixed forests (Augusto et al., 2002; Welp et al., 2017; Łuców, 2020). This difference in humidity levels could impact the migration and diffusion of dust, as higher relative humidity can lead to a shorter distance of dust diffusion (Zhou et al., 2021). Deciduous, mixed, coniferous forests and open canopy peatlands are four dominant ecosystem types in boreal regions which are anticipated to exhibit varying responses to the offsite effects of mines on the phyllosphere microbiota due to their distinct stand structures and physicochemical characteristics.

**FIGURE 1:**
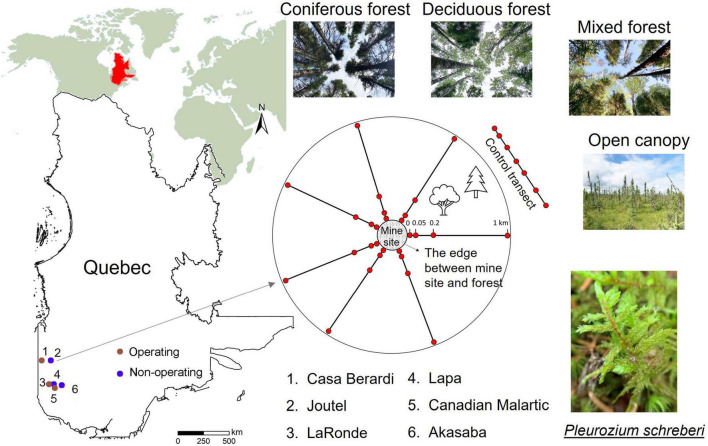
Study sites. **(Left)** Map of Quebec showing the location of the six gold mine sites. **(Right)** Sampling design. The four sampling plots at 0, 0.05, 0.2, and 1 km from the border of mine sites along transects at each study site are indicated by red circles. Six control transects were established near mine sites (see Supplementary Figure 1).

This study aimed to characterize the impact of mining on the feather moss phyllosphere microbiota in boreal landscapes and sought to answer the following questions: (Q1) do mine sites affect the feather moss phyllosphere microbiota offsite? (Q2) what is the main driver of the phyllosphere microbiota in mine offsite landscapes: ecosystem type, mining stage, or their interaction? (Q3) at what distance are offsite effects of mines on the phyllosphere microbiota detectable? To answer these questions, three operating mines and three non-operating sites were selected in the boreal forest of Quebec, Canada (Figure 1) and 1-km offsite landscapes around each one were used as study sites where *P. schreberi* shoots were collected to study their surface microbiata. Plots in mine offsite landscapes were classified in four ecosystem types (i.e., coniferous, deciduous, mixed forests and open canopy). These results will contribute to understanding the impact of mine sites on microbial communities in moss and identifying potential indicators of offsite mining effects.

## 2. Materials and methods

### 2.1. Study sites and design

To conduct the study, six mine sites were selected in the Abitibi-Témiscamingue and Nord-du-Québec regions of the province of Québec, Canada (Figure 1), including three operating mines (Casa Berardi, LaRonde and Canadian Malartic mine) and three non-operating sites (Akasaba, Joutel and Lapa mine sites). More detailed information about the six selected mine sites is available in Supplementary Table 1. Using a stratified selection process, 6−8 transects (the number of transects was selected based on the size of each mine area, see Supplementary Figure 1) were established perpendicular to each mine periphery. Four plots were established along each transect with distance from the edge of intact forest at 0, 0.05, 0.2, and 1 km. Furthermore, six control transects with 7−8 plots per-transect (44 plots in total) near the six mine sites were established in natural forests. The ecosystem of each plot was categorized into one of four types based on tree species [diameter at breast height (DBH) ≥10 cm] composition: coniferous forest (>70% of coniferous tree stems), deciduous forest (>70% deciduous tree stems), mixed (coniferous tree stems between 69 and 31%) and open canopy forests (DBH of all trees below 10 cm, peatlands in our studied areas). More detailed information about the design is shown in our previous studies (Boisvert et al., 2021; Yin et al., 2022).

### 2.2. Sampling

Between end of June and beginning of September of 2019, three colonies (i.e., a group of shoots of one species that live and interact closely with each other) of *P. schreberi* were randomly selected around the center point of each plot. The distance between colonies was approximately 10 m. At least 20 shoots per colony were picked, and debris and other plants were removed in the field. Only the top green parts (the last 2 years’ growth, 0−3 cm from the tip of each shoot, Street et al., 2013) of each individual shoot were collected to avoid contamination from soil particles. All samples from one plot were pooled in a sterile plastic roll bag and stored on ice in a cooler until reaching the laboratory, where samples were stored at −20°C until further processing. In total, 211 samples were collected from the 220 plots (no *P. schreberi* was found in 9 plots). In addition, forest environmental variables were also measured in the field as previously described (Yin et al., 2022). Principal component analysis (PCA) analysis showed that the environmental variables differed significantly among ecosystem types (ANOVA, *p* < 0.001, Supplementary Figure 2), therefore, environmental variables were grouped into the four ecosystem types in further analyses.

### 2.3. DNA library preparation and sequencing

The DNA extraction process followed the protocol of Kembel et al. (2014), with some modifications. 10−15 shoots were selected from the collected samples and placed into sterile 50 mL Falcon-tubes and phosphate buffered saline supplemented with Tween 20 (PBST) was poured into the tube to cover the shoots (30−35 ml). Samples were removed from the tube after shaking on vortex for 30 s and on a stirrer plate for 5 min. Microbial cells were recovered from the PBST by centrifuging at 4,000 × *g* for 20 min at 4°C. The supernatant was discarded and the pellet was transferred to a PowerBead tube of the DNeasy Power Soil DNA Isolation Kit (Qiagen, Valencia, CA, USA) for DNA extraction, following the manufacturer’s instructions. DNA extracts were quantified with the Qubit dsDNA HS assay kit using a Qubit Fluorometer 2.0 (Thermo Fisher Scientific, Watham, MA, USA). Bacterial and fungal communities were characterized by amplifying and sequencing the V4-V5 regions of the 16s ribosomal RNA gene with the primer pair 515F-Y (5′-GTGYCAGCMGCCGCGGTAA−3′)/926R (5′-CCGYCAATTYMTTTRAGTTT−3′) (Parada et al., 2016) and the ITS2 region of nuclear rRNA genes with the primer pair ITS9 (5′-GAACGCAGCRAAIIGYGA−3′) (Menkis et al., 2012)/ITS4 (5′-TCCTCCGCTTATTGATATGC−3′) (Bartels et al., 2018), respectively. Library preparation for Illumina sequencing was performed according to the manufacturer’s instructions for user-defined primers (Illumina, 2013). The first amplification was performed in a 25 μl reaction mix composed of 9 μl of UltraPure™ DNase/RNase-Free distilled water (Gibco, Thermo Fisher Scientific), 200 μM of each dNTP, 1.5 mM of Mg^2+^, 200 nM of each primer, 1 U of HotStarTaq Plus DNAPolymerase (Qiagen, Valencia, CA, USA), and 2.5 μl of DNA extract. Thermocycling conditions were as follows: initial denaturation step at 95°C for 5 min, 34 (bacteria) or 40 (fungi) cycles at 94°C for 30 s, 50°C for 30 s, and 72°C for 1 min, and a final elongation step at 72°C for 10 min. PCR products were purified with magnetic beads (Agencourt AMPure XP, Beckman Coulter, Mississauga, ON, CA, USA). Unique codes were added to each sample using the Nextera XT Index Kit following the manufacturer’s instructions (Illumina). Indexed amplicons were purified with magnetic beads, quantified with the Qubit dsDNA BR Assay Kit (Thermo Fisher Scientific) and pooled at equimolar concentration. Sequencing was performed on an Illumina MiSeq platform with a MiSeq Reagent Kit v3 (600 cycles) at the Next Generation Sequencing Platform of the Centre hospitalier universitaire de Québec-Université Laval Research Centre. Sequence data from this study are available at the NCBI Sequence Read Archive (SRA) under the BioProject PRJNA800026.

### 2.4. Bioinformatic processing

The DADA2 Pipeline (1.16 version for 16s and 1.8 version for ITS, Callahan et al., 2016) on R platform (R Core Team, 2021) was used to exclude primer sequences, filter and de-noise sequences, de-replicate unique amplicon sequence variants (ASVs, similar to 100%-identity operational taxonomic units), and remove chimeric sequences. Those ASVs were classified (minBoot = 80) with the SILVA 138 (Quast et al., 2013) and UNITE 8.2 (Koljalg et al., 2005) taxonomic databases for 16 s and ITS identification, respectively. Then, we identified and removed non-bacterial and non-fungal ASVs from bacterial and fungal ASV tables including ASVs classified as Archaea, Mitochondria, Chloroplast, Eukaryote and Rickettsioses. After the processing, 38,901 and 20,086 ASVs remained for bacteria and fungi, respectively.

The tables generated by the DADA2 Pipeline were imported into R (3.4) as a phyloseq object (*phyloseq* package version 1.12.3) (McMurdie and Holmes, 2013) for further quality filtering steps. The removal of sequences identified as contaminants by the *decontam* R package (Davis et al., 2018) was used, and then singletons, doubletons and ASV with less than 10 reads were removed to control for the potential influence of rare sequences. One sample from the LaRonde site showed an unusual and much higher fungal richness than other samples based on visual inspection of rarefaction curves and was removed from further analyses. To analyze the alpha diversity of the phyllosphere microbiota, the datasets were rarefied to the minimum sample size (2,383 reads per sample for bacteria and 6,153 reads per sample for fungi, Weiss et al., 2017), whereas, for community similarity analysis, library size normalization was carried out using the geometric mean of pairwise ratios (Chen et al., 2018).

### 2.5. Statistical analyses

To answer the three specific questions identified in the sections “1. Introduction” (Q1–Q3) and “2.5. Statistical analyses” were divided in two sections: (1) examine offsite effects of mines on the diversity, community structure and composition of the bacterial and fungal communities of the phyllosphere and identify drivers (ecosystem type and mine stage) of these communities (Q1 and Q2); (2) determine the distance influenced by offsite effects (Q3). All analyses were performed with the statistical platform R 4.0.5 (2021-03-31) with R Studio software. Results were visualized with the *ggplot2* package version 2.0.0 (Wickham, 2011).

#### 2.5.1. Effects of ecosystem type and mine stage on the phyllosphere microbiota

To test the presence of offsite effects of mines on the phyllosphere microbiota (Q1) and identify potential drivers of microbial diversity, community structure and composition (Q2), alpha, beta diversity and relative abundance of individual taxa were analyzed. For alpha diversity analysis, generalized linear mixed models (GLMMs, “glmer” function from the *lme4* R package version 1.1-23, Bates et al., 2018) were applied on mining stage, ecosystem type and their interaction as predictors. Considering the nested structure of the sampling design, the nested terms site, transect and plot were included as random effects. Observed richness (number of ASVs), Shannon and InvSimpson indexes (Shannon and InvSimpson indexes were calculated with “estimate_richness” function from *phyloseq* package) were selected as the response variables for bacterial and fungal alpha diversity, separately. Based on the inherent statistical characteristics of response variables, Negative Binomial (overdispersed count data, observed bacterial and fungal richness, with “glmer.nb” function in *lme4* R package) and Gaussian (continuous number with normally distributed residuals, Shannon and InvSimpson Indexes with “glmer” function with family = gaussian and a log link) distribution were used in the models. Marginal R squared of GLMMs were calculated with the function “r.squaredGLMM” of the *MuMIn* R package R package 1.15.6 (Barton and Barton, 2015). Significant *p*-values for the effects of mining stage, ecosystem type and their interaction were calculated with ANOVA using Type II sum of squares [Type II sum of squares is more powerful than Type III for ANOVAs with unbalanced data, Langsrud, 2003, “Anova” function in the package “car” version 3.0-2 (Fox et al., 2019)]. When interaction terms were not significant, they were removed from the final models. When the interaction term was not significant, multiple pairwise comparisons were conducted across mine stages and ecosystem types. When the interaction term was significant, comparisons were made between mine stages within each ecosystem type. When the interaction was not significant, pairwise comparisons between each mine stage and between each ecosystem type, respectively, were conducted with TukeyHSD test (“*emmeans*” function in the *emmeans* package version 1.3. 5.1, Lenth et al., 2019).

Similarly, the effects of ecosystem type and mining stage on phyllosphere beta diversity were tested by permutational multivariate analysis of variance (PERMANOVA, “adonis” function from the *vegan* package version 2, Oksanen et al., 2013) on Bray-Curtis dissimilarity matrices (“vegdist” function in the *vegan* package) with 999 permutations. PERMANOVA tests were performed for the bacterial and fungal ASV matrices as the response and ecosystem type, mining stage and their interaction as the explanatory variables and site factor as a “strata” term to reduce randomizations across all sites. Bray-Curtis distance matrices were further visualized using non-metric Multi-dimensional Scaling (NMDS, “metaMDS” function in the *vegan* package) with three dimensions. We also used *post hoc* pairwise PERMANOVAs (999 permutations, “pairwise.adonis” function in the package *RVAidememoire* version 0.9-45-2, Hervé, 2014) with a multiple comparison correction based on Benjamini–Hochberg method (Benjamini and Hochberg, 1995) to compare differences between mining stages.

Then, relative abundance of main bacterial and fungal phyla (average relative abundance across all samples >0.5%) was used as response variables to determine the effects of mining stage and ecosystem type (similar analysis process as for alpha diversity). Microbiome relative abundances are compositional data that range between 0 and 1 and are also generally zero-inflated (Peng et al., 2016; Ho et al., 2019), therefore zero-inflated beta mixed models (ZIBMMs, and “glmmTMB” function with ziformula = ∼ and family = beta_family in *glmmTMB* package version 0.1.3, Brooks et al., 2017) were used instead of GLMMs here. The models that showed convergence errors were corrected by optimization of the model algorithm *via* Broyden–Fletcher–Goldfarb–Shanno (BFGS) algorithm (Dai, 2002) method in the function of the “glmmTMBControl” from *glmmTMB* package. Marginal R squared of ZIBMMs were calculated with the function “r2” of the *sjmisc* package version 2.4.0 (Lüdecke and Lüdecke, 2019). Furthermore, to identify the taxa (ASVs) driving differences in community composition between offsite landscapes of operating, non-operating mine sites and controls, indicator species analysis (“multipatt” function in the *indicspecies* package version 1.7. 6, De Caceres et al., 2016) with point biserial-correlation coefficient (phi) was applied.

#### 2.5.2. Effects of the distance from mines on the phyllosphere microbiota

The effects of distance from the mines on alpha and beta diversity of bacterial and fungal communities (Q3) and on the relative abundance of individual taxa (i.e., main phyla, top 10 main genera through all samples, and common ASVs found in at least 90% of samples for bacteria and 70% of samples for fungi) were analyzed. Control transects were removed from these analyses given that the control transects were designed for landscape-level analysis (Q1 and Q2). Firstly, to assess variations in diversity and community structure (alpha and beta diversity) along the transects, alpha diversity indices (Observed ASV richness, Shannon and InvSimpson) and Bray–Curtis dissimilarity matrices were used as response variables in GLMMs and Pairwise PERMANOVAs, respectively. The effect of distance and its interaction with mining stage (only operating and non-operating here) were used as predictors. The analysis process (error distribution, ANOVA test and *post-hoc* test) was similar to the models we used in Q1 and Q2, but here transects were nested into sites, and ecosystem type were used as random factors. Distance influenced by offsite effects of mines were determined based on the results of TukeyHSD tests and graphs (showing the position of threshold point, “ggplot” function in *ggplot2* package). Considering the very large ranges of variation (orders of magnitude) between taxa and zeros in relative abundance datasets, row data were replaced by log (x + l) transformation to present the spatial pattern with the distance in graphs. Finally, the offsite footprint of mines was evaluated and estimated using the influenced distance for alpha and beta diversity of bacterial and fungal communities as well as the relative abundance of individual taxa.

## 3. Results

### 3.1. Effects of mining stage and ecosystem type on the phyllosphere microbiota

**FIGURE 2:**
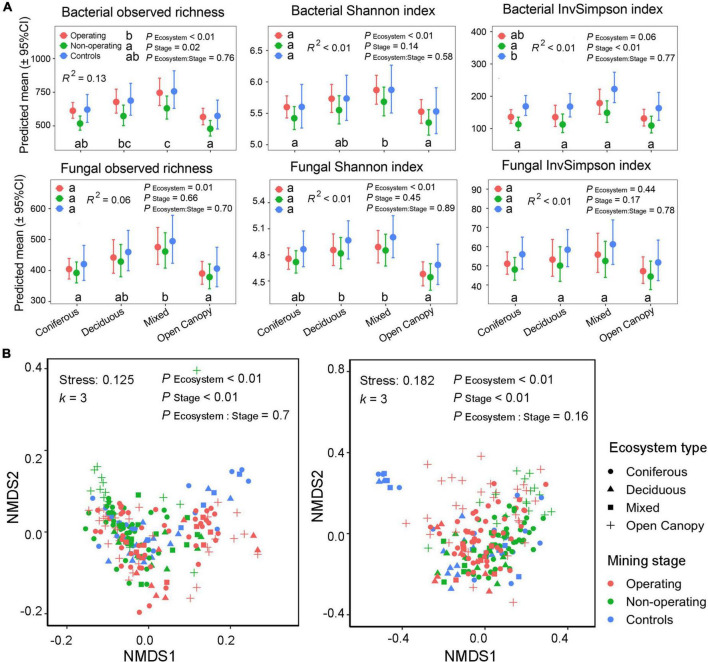
Changes in the phyllosphere alpha and beta diversity in mine offsite landscapes. **(A)** Alpha diversity for both bacterial and fungal communities at different mining stages (plots surrounding operating sites, *N* = 92; plots surrounding non-operating sites, *N* = 78; plots in controls, *N* = 40) in four ecosystem types (coniferous forest, *N* = 101; deciduous forest, *N* = 33; mixed forest, *N* = 26; open canopy, *N* = 50). Points show means for all samples; bars show a 95% confidence interval around the mean. Different lowercase letters indicate significantly different means across mining stage and ecosystem type, respectively. *P*-value based on generalized linear mixed models (type II sum of squares) and Tukey HSD pairwise comparisons (α = 0.05) were used to test the differences across ecosystem types and mining stages, respectively. **(B)** Beta diversity of bacterial and fungal community structure. Non-metric Multi-dimensional Scaling (NMDS) are based on Bray–Curtis dissimilarity. Tests in the top side of the panels present the results of PERMANOVA.

Mining stage and ecosystem type both affected *P. schreberi* phyllosphere alpha and beta diversity in mine offsite landscapes without interaction between the two factors. Bacterial alpha diversity differed among mining stages (observed richness and InvSimpson index, ANOVA type II sums, *P*_Stage_ < 0.05, Figure 2A). No significant differences in alpha diversity were found between operating sites and controls (*post-hoc* test results in Figure 2A), but both had higher alpha diversity values than non-operating sites (Figure 2A). The effects of mining stage on bacterial alpha diversity did not depend on ecosystem type (ANOVA type II sums, *P*_Ecosystem:Stage_ > 0.05, Figure 2A), but ecosystem type also affected the alpha diversity (bacterial observed richness and Shannon index, ANOVA type II sums, *P*_Ecosystem_ < 0.05, Figure 2A). Generally, deciduous and mixed forests had a higher alpha diversity than coniferous forests and open canopy in the offsite landscapes (bacterial observed richness and Shannon index, Figure 2A). Similar patterns were found in fungal alpha diversity, with higher alpha diversity found in deciduous and mixed forests (fungal observed richness and Shannon index, Figure 2A) and the lack of interaction between mining stage and ecosystem type. But in contrast to bacteria, none of the fungal diversity indices measured was significantly affected by the mining stage (ANOVA type II sums, *P*_Stage_ > 0.05, Figure 2A). Furthermore, only 0−13% of shifts in bacterial and fungal alpha diversity were explained by fixed factors (mining stage and ecosystem type) in the models (see *R*^2^ in Figure 2A).

Similarly, the analysis of the beta diversity of phyllosphere bacterial and fungal communities showed a significant effect of both the mining stage (bacteria, *R*^2^ = 4.43%, fungi, *R*^2^ = 2.62%, PERMANOVAs in Figure 2B and Supplementary Table 2) and ecosystem type (bacteria, *R*^2^ = 5.83%, fungi, *R*^2^ = 4.63%, PERMANOVAs in Supplementary Table 2) without interaction between the two factors (PERMANOVAs, *P*_Ecosystem:Stage_ > 0.05, Figure 2B). Furthermore, all pairwise comparisons for mining stage and ecosystem type showed significant differences in bacterial and fungal community structure (Pairwise PERMANOVA tests, *P* < 0.01, Supplementary Tables 3, 4).

**FIGURE 3:**
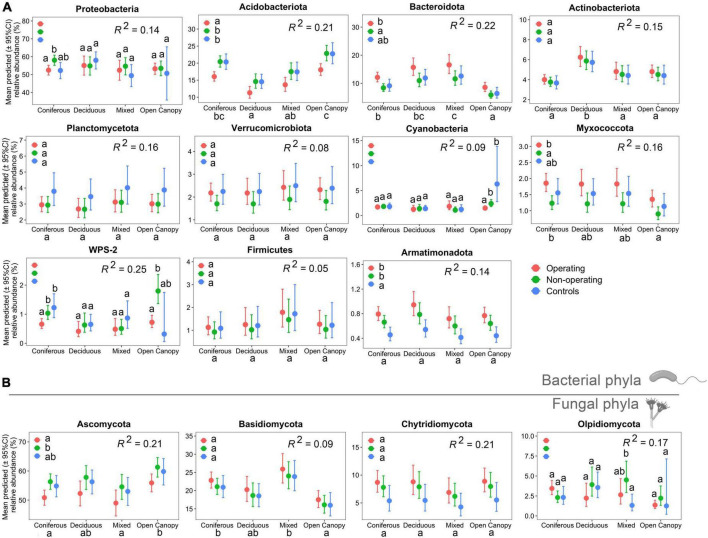
Differences in relative abundance of main phyllosphere phyla (relative abundance >0.5%) at each mining stage in four ecosystem types in mine offsite landscapes. Zero-inflated beta regression models were used to determine responses of relative abundance of main bacterial **(A)** and fungal **(B)** phyla to ecosystem type, mining stage and their interaction. Points show means for all samples; bars show a 95% confidence interval around the mean. Different lowercase letters indicate significantly different means across groups based on Tukey HSD pairwise comparisons (α = 0.05). When interaction between ecosystem type and mining stage was significant, pairwise comparisons were used between mine stage in each ecosystem type, while when the interaction was not significant, the interaction term was removed from the models and pairwise comparisons were used between ecosystem type and mining stage, separately.

Mining stage and ecosystem type both had a significant effect on the relative abundance of most phyla detected in the moss phyllosphere. The effect of mining stage was variable depending on the phylum. The relative abundance of the bacterial phylum Acidobacteriota and the fungal phylum Ascomycota were lower in offsite landscapes of operating sites than in non-operating sites across all samples (see Tukey HSD pairwise comparisons in Figure 3), while the relative abundances of the bacterial phyla Bacteroidota and Myxococcota were higher near operating sites than non-operating sites (Figure 3). The relative abundance of the Armatimonadota was higher in both operating and non-operating sites than in the controls (Figure 3). The relative abundance of dominant phyla also differed between ecosystem types. Higher relative abundance of Acidobacteriota was detected in coniferous forest and open canopy ecosystems than in deciduous and mixed forests, while deciduous and mixed forests had a higher relative abundance of Bacteroidota. A significant interaction effect between the mining stage and ecosystem type on the relative abundance of four bacterial phyla (i.e., Proteobacteria, Cyanobacteria, WPS-2 and Olpidiomycota, Figure 3) was observed. Overall, 5−25% of the shifts in the relative abundance of bacterial and fungal phyla were explained by fixed factors (mining stage and ecosystem type) in the models (see *R*^2^ in Figure 3).

**FIGURE 4:**
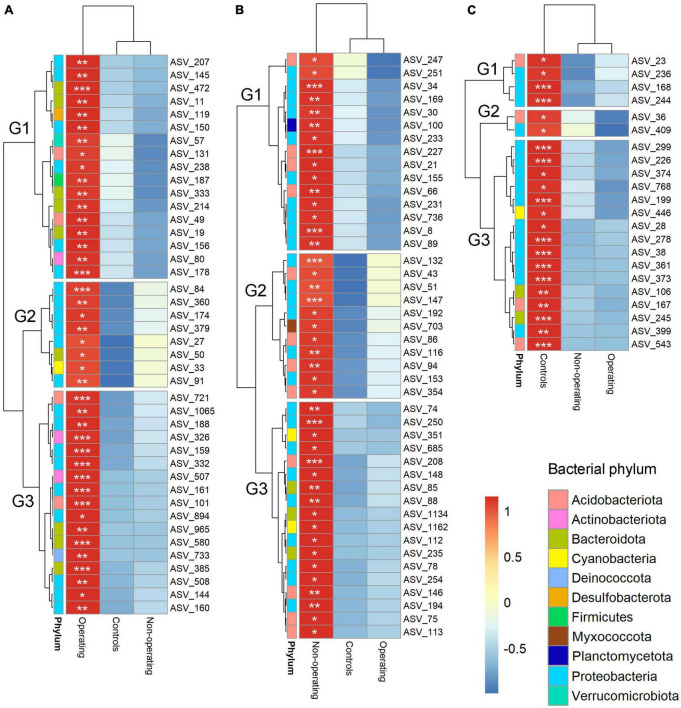
Hierarchical clustering heatmaps of the relative abundance (scale by row) of bacterial indicator ASVs for mining stages. **(A)** Indicators for operating mines; **(B)** indicators for non-operating sites; and **(C)** indicators for control sites. The color-coded scale indicates an increase (red) and a decrease (blue). Only ASVs with total relative abundance >0.05% were included in the indicator analysis. *P < 0.05; **P < 0.01; ***P < 0.001. Note relative abundance of each bacterial and fungal ASV is shown for each mine stage when not an indicator ASV in that stage.

**FIGURE 5:**
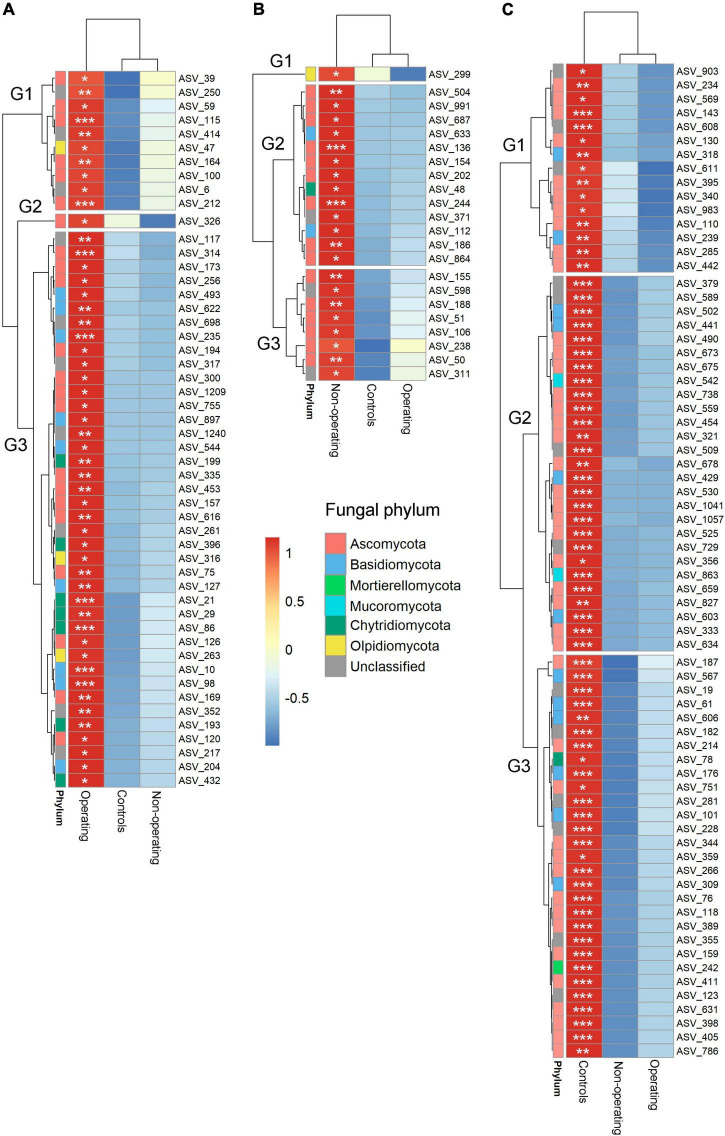
Hierarchical clustering heatmaps of the relative abundance (scale by row) of fungal indicator ASVs for mining stages. **(A)** Indicators for operating mines; **(B)** indicators for non-operating sites; and **(C)** indicators for control sites. The color-coded scale indicates an increase (red) and a decrease (blue). Only ASVs with total relative abundance >0.05% were included in the indicator analysis. *P < 0.05; **P < 0.01; ***P < 0.001. Note relative abundance of each bacterial and fungal ASV is shown for each mine stage when not an indicator ASV in that stage.

Indicator species analysis was performed to identify fungal and bacterial taxa driving the differences in microbial communities based on mining stage. Phyllosphere of *P. schreberi* near operating sites, non-operating sites and control sites were characterized, respectively, by 42 (8 phyla), 44 (6 phyla) and 22 (4 phyla, Figure 4 and Supplementary Table 6) bacterial indicator species, and 54 (4 phyla), 23 (4 phyla) and 78 (5 phyla) fungal indicator species (Figure 5 and Supplementary Table 7) (in this case, the term “species” refers to ASVs). Bacterial ASV_84 (stat = 0.318, Supplementary Table 6) and ASV_101 (stat = 0.318, Supplementary Table 6) assigned to the genera *Acidiphilium* and Candidatus *Solibacter*, respectively, were the best indicator species for operating sites (Supplementary Table 6), whereas bacterial ASV_8 (stat = 0.279, family Acetobacteraceae, Supplementary Table 6) and ASV_361 (stat = 0.514, genus *Cupriavidus*, Supplementary Table 6) were the best indicators of non-operating sites and controls, respectively, (Supplementary Table 6). Fungal ASV_10 (stat = 0.326, genus *Phenoliferia*, Supplementary Table 7) was the best indicator species for operating mines, while fungal ASV_136 (stat = 0.314, order Orbiliales, Supplementary Table 7) and ASV_429 (stat = 0.397, genus *Thelephora*, Supplementary Table 7) were the best indicator species of non-operating sites and controls, respectively.

### 3.2. Effects of distance from the mine on the phyllosphere and offsite footprint

**FIGURE 6:**
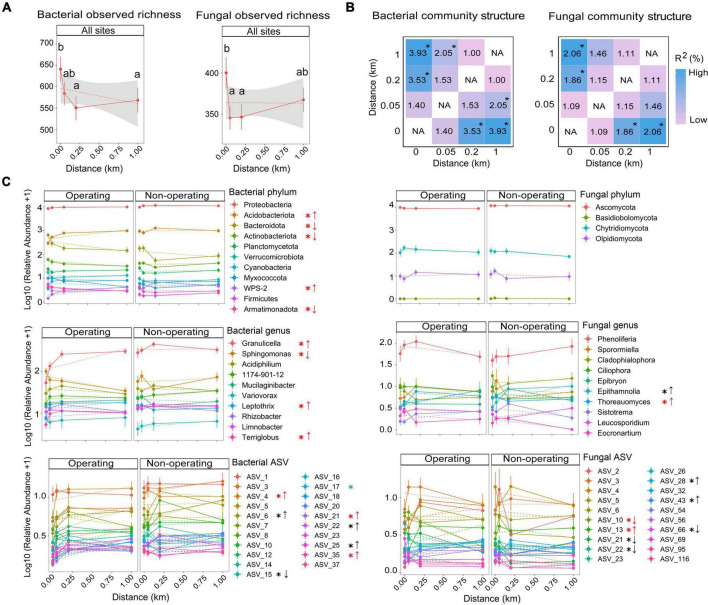
Changes in phyllosphere microbial communities with distance from mine sites. **(A)** Bacterial and fungal observed richness. Different lowercase letters indicate significantly different means between different distances at 0 (*N* = 39), 0.05 (*N* = 44), 0.2 (*N* = 43), and 1 km (*N* = 44) from mine sites (results based on generalized linear mixed models and Tukey HSD pairwise comparisons, α = 0.05). **(B)** Bacterial and fungal community structure differences between distances based on the results of pairwise PERMANOVA on Bray-Curtis dissimilarity matrices, *P < 0.05. **(C)** Relative abundance of bacterial and fungal taxa along the distance at phylum, genus and ASV levels. Points show means for relative abundance after log (x + l) transformation; bars show standard error (SE). Black asterisks indicate significant association with distance at both mining stages based on the zero-inflated beta mixed regression models and *post-hoc* tests with Tukey HSD pairwise comparisons. Red asterisks indicate the interaction between the distance and mining stage was significant and the effects occurred near operating mines, while green asterisks indicate the effects occurred near non-operating sites. Arrows used for linear patterns: up arrows, relative abundance is increasing with the distance; down arrows relative abundance is decreasing with the distance.

Phyllosphere alpha and beta diversity were associated with the distance from mines without interaction with the mining stage (ANOVA type II sums for GLMMs in bacterial and fungal observed richness and Shannon indices, *P*_Distance_ < 0.05, *P*_*Stage*_ < 0.05 and *P*_Distance:Stage_ > 0.05; PERMANOVAs in bacteria and fungi, *P*_Distance_ < 0.001, *P*_Stage_ < 0.001 and *P*_Distance:Stage_ > 0.05, Supplementary Tables 8, 9). Bacterial alpha diversity (observed richness and Shannon index) showed a decreasing trend with the distance from mine sites. Observed richness and Shannon index were, respectively, about 13% and 4% higher at 0 km than at 1 km, although only the 0 km value was significantly higher compared to the 0.2 and 1 km values (TukeyHSD test results in Figure 6 and Supplementary Table 8). In contrast, fungal alpha diversity (observed richness and Shannon index) followed a non-linear pattern with the distance, with no significant difference between 0 and 1 km but with significant differences between 0 and 0.05 or 0.2 km (TukeyHSD tests in Figure 6 and Supplementary Table 8). For beta diversity, bacterial community structure at 0 and 0.05 km differed from that at 1 km (Figure 6B), while fungal community structure differed between 0 and 1 km, but no significant difference was found between 0.05 and 1 km (Figure 6B). Therefore, in most cases, the variation of phyllosphere alpha and beta diversity was only detectable until 0.05 km into the forest interior.

In contrast to alpha and beta diversity results, analysis of the relative abundance of individual taxa detected significant interactions between the distance and the mining stage as well as significant effects of mining up to over 0.2 km. In total, for five phyla, four genera and four ASVs among bacterial taxa, as well as one fungal phylum and two fungal ASVs, the effect of distance on the relative abundance based on GLMMs depended on whether the mine site was operating (Figure 6C and Supplementary Tables 10-12). Relative abundance of three bacterial phyla (i.e., Bacteroidota, Actinobacteriota, and Armatimonadota), one bacterial genus (i.e., *Sphingomonas*) and one fungal ASV (ASV 10, *Phenoliferia* see Supplementary Table 8) were positively influenced by mines, with highest values (between 1.4 and 3.14-fold higher than at 1 km) detected at 0 km and decreasing with the distance near operating mines. No linear associations with distance were found near non-operating sites for these taxa (Figure 6C). For another four ASVs (bacterial ASV 15, *Sphingomonas*, and fungal ASV 21, Spizellomycetales order, ASV 22, *Cladosporium*, ASV 66, *Aureobasidium*, Figure 6C and Supplementary Table 12), the relative abundance was positively affected by mines near both operating and non-operating sites (1.63−7-fold changes). Furthermore, two bacterial phyla (i.e., Acidobacteriota and WPS-2), three bacterial genera (i.e., *Granulicella*, *Leptothrix*, and *Terriglobus*) and six bacterial ASVs, as well as two fungal genera (i.e., *Epithamnolia* and *Thoreauomyces*) and four fungal ASVs were negatively affected by either operating mines or all mine sites (both operating and non-operating, Figure 6C).

## 4. Discussion

### 4.1. The presence of offsite effects of mines on the feather moss phyllosphere microbiota (Q1, Q2)

Our results confirmed the presence of offsite effects of mines on the feather moss phyllosphere microbiota (Q1) based on the differences in alpha and beta diversity, relative abundance of individual taxa, and indicator species of mined sites (operating or non-operating) and controls. These findings extend the offsite effects of mines on surrounding ecosystems to microbiomes, as only vegetation and animals were used to determine the effects in previous studies (Dyer et al., 2001; Boisvert et al., 2021; Watkinson et al., 2021; Wu et al., 2021; Yin et al., 2022). Surprisingly, similar levels of bacterial alpha diversity were detected in operating mines and controls, while the diversity in non-operating sites was lower (Figure 2), which is contrary to our expectations. Integrating these results with those obtained for individual taxa, we suggest that this result is explained by taxon-specific responses to offsite effects. Some taxa (e.g., Armatimonadota, Figure 3) were favored near mine sites, although some other (e.g., Acidobacteriota, Figure 3) were suppressed, leading to similar levels of diversity as in the control sites. These results seem to support the novel “niche flip” mechanism in microbial ecology, where alpha diversity is shaped by both disturbance frequency and intensity (Mancuso et al., 2021). In this mechanism, the microbial communities follow a U-shaped diversity dependence on the disturbance intensity, with the lowest level of diversity occurring at an intermediate level of disturbance (Mancuso et al., 2021). Here, controls, non-operating and operating sites represent zero, low and intermediate mining disturbance intensity, respectively, and followed this high-low-high (U-shaped) pattern in alpha diversity. Further work will be needed to confirm the mechanisms underlying these observations, especially regarding the fluctuation and intensity of the offsite effects, which were not characterized in this study.

Differences in phyllosphere fungal and bacterial beta diversity between mining stages confirmed that the mining stage is a driver of microbial community structure in mine offsite landscapes. This can be attributable to the fact that mine production directly determines dust production (Wang et al., 2022). Previous studies have identified pH, total organic carbon, total phosphorus and heavy metal content as the main influencing factors in the variation of soil microbial community structure in mining polluted areas (Dimitriu and Grayston, 2010; Kane et al., 2020; Signorini et al., 2022; Wu et al., 2022). These factors could also partly explain the variation observed in the moss phyllosphere in this study considering that feather moss grows in close contact with the soil. The concentration of metals (aluminum, copper) was also significantly correlated with variations in microbial functions in both phyllosphere and soil study comparing the phyllosphere microbiota of various tree species (Lajoie et al., 2020). Considering that metal concentrations delivered by the dust could be higher in the phyllosphere near operating than non-operating mines and that mosses are known to accumulate metals (Blagnytë and Paliulis, 2010; Stankoviæ et al., 2018), we can expect that metal concentration in the moss phyllosphere could be responsible for the differences in community structure observed here between mining stages. For example, the drastic decline of Acidobacteriota near operating mines compared with non-operating and control sites indicated the sensitivity of Acidobacteria to heavy metals (Macdonald et al., 2011; Guo et al., 2019; Zheng et al., 2019). In contrast, the higher relative abundances of Bacteroidota near operating mines suggest that these landscapes could provide favorable conditions for some microorganisms. Bacteroidota usually harbor the majority of heavy metal resistance genes (Yan et al., 2020), and is able to quickly adapt and endure high heavy metal stresses in mining polluted areas (Zhao et al., 2020a,b; Zou et al., 2021). Characterizing soil properties, assessing dust emissions from mines, and measuring heavy metal concentrations in mosses would be important next steps to further identify the environmental drivers responsible for changes in phyllosphere community structure observed in mine offsite landscapes.

Furthermore, operating gold mines supported some specific species, as highlighted by the indicator species analysis. Bacterial ASVs 101 and 84 as well as the fungal ASV 10 were the best indicators of operating gold mines (Supplementary Tables 6, 7) and belonged to Candidatus *Solibacter, Acidiphilium* and *Phenoliferia*, respectively. The ability of members from the first two bacterial taxa to overcome stressful conditions (including high metal concentrations) have been reported in mining environments of other minerals (Böhmer et al., 2020; Li L. et al., 2020). Furthermore, members of the fungal genus *Phenoliferia* can degrade phenolic compounds (Perini et al., 2019; Pakarinen et al., 2021) and its higher relative abundance near operating mines might be driven by a higher phenolic compounds content of feather moss, as these compounds are usually produced by plants as a defense mechanism under heavy metal stress (Maleki et al., 2017). More studies are needed to explore which environmental variable or plant traits (such as heavy metals and phenolic compounds content) affected by gold mining activities lead to the increased relative abundance of Fungal ASV 10 (*Phenoliferia*) near mines. Nevertheless, fungal ASV 10, with its relatively high abundance (about 1.5% at 0 m near operating mines, Supplementary Table 12) and its sensitivity to mining stage and distance, could be a reliable indicator to assess the presence and magnitude of offsite effects of the gold mines. Surprisingly, one of the best indicator ASV for control sites (bacterial ASV361) was assigned to the *Cupriavidus* genus, which contains several species or strains known for their heavy metal resistance and adaptation to metal-contaminated environments (Reith et al., 2009; Mijnendonckx et al., 2013; Van Houdt et al., 2018). The species or strain identified here in the moss phyllosphere may lack the specific genes required for heavy metal resistance, which could be confirmed by further characterizing the moss phyllosphere DNA using shotgun metagenomics approaches.

Our results confirmed that offsite effects are usually cryptic because inherent limitations of impact evaluation (e.g., experimental design, detection methods, spatial scales, statistical power) led to those effects being overlooked (Raiter et al., 2014). If our experimental design had included operating mines only, and if the focus of our statistical analyses had been limited to alpha diversity, substantial offsite effects could have been undetected because taxa-specific responses were masked in the alpha diversity response (especially for fungi, Figure 2). This study also highlights that the use of environmental DNA (eDNA) tools can reveal impacts of disturbances on groups of living organisms (here bacteria and fungi, but many others could be targeted) that are typically overlooked in impact evaluations because of the absence of efficient methods to detect them. The performance of these tools to assess biodiversity and measure the environmental impacts of disturbances is becoming widely accepted by the scientific community (Edge et al., 2020; Pawlowski et al., 2021) and hopefully, this will lead to a wider application of these tools in impact assessment in the near future.

### 4.2. Ecosystem type and mining stage affected phyllosphere microbiota in mine offsite landscapes (Q2)

Our results provide further evidences that feather moss phyllosphere diversity and composition differed between ecosystem types (Jean et al., 2020b; Rodríguez-Rodríguez et al., 2022). However, no differences in alpha diversity (bacterial and fungal observed richness and bacterial Shannon index, Figure 2B) were found between deciduous and coniferous forests in contrast with Rodríguez-Rodríguez et al. (2022) who found higher bacterial alpha diversity of the feather moss phyllosphere in coniferous stands (black spruce) than in deciduous stands (trembling aspen). To avoid a loss of statistical power by creating too many categories, coniferous forests and deciduous forests in this study were not monospecific stands, which may have led to these differences. Our observations of a higher relative abundance of the bacterial phylum Acidobacteria in coniferous forests and open canopy and a higher relative abundance of Bacteroidota in deciduous and mixed forests were, however, consistent with findings from Rodríguez-Rodríguez et al. (2022). Despite the limitation that most forest stands in this study were located near mine sites and not in natural conditions, our results still provide insights into the relationships between ecosystem types and moss phyllosphere communities in boreal forests.

Surprisingly, the effects of mining stage on the alpha, beta diversity and relative abundance of most phyla were not ecosystem type-dependent. It suggests that ecosystem types did not influence the ability of the moss phyllosphere to resist to the offsite effects, which is in contrast with a previous study indicating that bryophyte diversity was more affected by mining stage in deciduous forest than in coniferous forest (Yin et al., 2022). Compared with deciduous forest, coniferous trees with high canopy cover, leaf area index and persistent foliage intercept more pollutants in atmospheric deposition (Barbier et al., 2008; Nguyen et al., 2015). Here, this mechanism did not play an obvious role in reducing the impact of mines on the moss phyllosphere microbiota as the effects were similar in coniferous and deciduous forests. To our knowledge, no study to date has directly compared the sensitivity of bryophyte communities to that of phyllosphere microbial communities to dust, but microorganisms colonizing the leaf surface are know to be strongly exposed to adverse conditions, including air pollutants (Bringel and Couée, 2015), and have been found to be more sensitive to environmental conditions than leaf endophytes (Sivakumar et al., 2020). A study also found that the overall reduction of dustfall by coniferous trees was between 38 and 42%, while deciduous trees reduced dustfall by 27−30% (Dochinger, 1980). So over 50% of dustfall still could pass through the canopy structure, leading to loads of heavy metals and other toxins that may have impacted the phyllosphere microbial communities without reaching, in coniferous forests, the level at which bryophyte diversity would also be significantly impacted. On the other hand, it is also possible that an interaction between ecosystem types and mining stage would have emerged if the phyllosphere was sampled later in the season after leaf fall, when the differences in canopy structure were more striking than in the summer. Further studies should be done to examine these two potential explanations. Overall, the results indicate that predicted shifts in ecosystem types should not generally modify the offsite effects of mines on the phyllosphere microbiota in boreal landscapes.

We also found that fungal communities were more resistant to the offsite effects than bacterial communities. According to previous studies, fungi appear to be more tolerant to heavy metals than bacteria (Kelly et al., 2003; Zeng et al., 2020), and exposure to heavy metals in the mine environment may have triggered additional evolutionary adaptation in the fungal communities (Adriaensen et al., 2005).

### 4.3. The footprint of offsite effects of mines on feather moss phyllosphere (Q3)

Determining the offsite footprint of mines is challenging but necessary for policy maker and governments to assess ecological impacts of mining projects and, subsequently, develop mitigation strategies. Our findings indicated that phyllosphere alpha and beta diversity were affected by the distance from mine site, but the effect was only detectable up to a distance between 0 and 0.05 km from the edge of mine sites, which is much lower than what was detected using the relative abundance of individual taxa (up to 1 km, such as fungal ASV10 near operating mines, Supplementary Table 12). Therefore, if the offsite footprint is determined at the community-level, sizes of the offsite footprint and their impacts on ecological services could be underestimated.

Based on all results of alpha, beta diversity and relative abundance of individual taxa, we found that significant effects are detectable within 0.2 km of mine sites for most taxa, especially for operating mines. Therefore, if the average width of the offsite footprint surrounding mines is set to 0.2 km, the total area for footprint calculation would be about 4.5 km^2^ (average 1.5 km^2^/mine, measured through Google Earth Pro 7.1) for the three operating mines in our study. With more than 6,000 active mines across the globe (Maus et al., 2020), the estimated global offsite footprint of active mines on surrounding phyllosphere microbiota could be up to 9,000 km^2^ (about the land area of Cyprus). Considering non-operating sites still have effects on surrounding phyllosphere structure, the actual offsite effects of mines could be even larger. Although the mining method, size, shape and local environmental conditions could affect the range of offsite effects, our results still provide justification to include offsite footprints of mines when evaluating the total footprint of mining on landscapes.

## 5. Conclusion

Our findings highlight the presence of offsite effects of mines on microbiomes, as exemplified here in the feather moss phyllosphere. Although both mining lifecycle and forest composition were identified as factors affecting the offsite effects of mines, our study indicates that these effects are cryptic and can easily be ignored at the community level. Specific taxa such as Bacteroidota and fungal ASV10 (*Phenoliferia*) have higher indicator roles than community-level diversity to determine the presence and footprint of offsite effects of mines. Though the average distance influenced by the offsite effects is small (about 0.2 km) near individual mines, the sum might be large and impactful across the globe. Moreover, as more mining projects will be established in the coming decades with the energy transition, more landscapes will be exposed to the offsite effects of mines. The global offsite footprint of mines is therefore expected to expand and become a problem over larger areas. Further studies are needed to assess how these changes in microbial communities of the phyllosphere in mine offsite landscapes affect ecosystem functioning, including carbon and nitrogen cycling in boreal ecosystems, and to develop appropriate mitigation strategies.

## Data availability statement

The data presented in this study are deposited in the NCBI-SRA (Sequence Read Archive) repository, accession number PRJNA800026. The data is available in Genbanck (https://www.ncbi.nlm.nih.gov/search/all/?term=PRJNA800026).

## Author contributions

XY, CM, and NF contributed to conception and design of the study. XY organized the database and wrote the first draft of the manuscript. XY and AS performed the statistical analysis. All authors contributed to manuscript revision, read, and approved the submitted version.
