# Supplementary material for: Out of site, out of mind: Changes in feather moss phyllosphere microbiota in mine offsite boreal landscapes
Source: Front Microbiol. 2023 Apr 5;14:1148157. doi: 10.3389/fmicb.2023.1148157 (PMC10113616; doi:10.3389/fmicb.2023.1148157)
Supplement: Supplementary file 1 [file Data_Sheet_1.docx]

**SUPPLEMENTARY MATERIALS**

**Table S1.** Information about the selected respective mines

|  | **Akasaba** | **Canadian Malartic** | **Casa Berardi** | **LaRonde** | **Lapa** | **Joutel** |
| --- | --- | --- | --- | --- | --- | --- |
| **Stage** | Establishing | Operation | Operation | Operation | Closing | Restored |
| **Main disturbances** | Roads, deforestation and some human activities | Activities associated with mining (e.g., blasting, digging and transportation) | | | Residual contaminants and some human activities | Residual contaminants (e.g., tailings) |
| **Mine type** | Open Pit | Open Pit | Underground & Open Pit | Underground | Underground | Open pit |
| **Commodities** | Gold, Copper | Gold, Silver | Gold, Silver | Gold, Silver, Copper, Zinc | Gold | Gold, Copper |
| **Owned/operated by** | Agnico Eagle Mines Ltd. | Canadian Malartic Corp. | Hecla Quebec Inc. | Agnico Eagle Mines Ltd | Agnico Eagle Mines Ltd | Agnico Eagle Mines Ltd |
| **Num. Employees** | - | 699 (2017) | 580 (2018) | 833 (2017) | 165 (2018) | - |
| **Mine life** | Preparation since 2014 | Operation since 2005 | Operation since 1988 | Operation since 1988 | Closed since2018 | Closed since 1988 |
| **Mining Method** | - | ▪ Truck & Shovel/ Loader | ▪ Truck & Shovel/ Loader  ▪ Longhole stoping  ▪Timbered stoping  ▪ Longitudinal stoping | ▪ Transverse open stoping  ▪ Longitudinal retreat  ▪ Cemented backfill  ▪ Paste backfill | ▪ Transverse open stoping  ▪ Longitudinal retreat  ▪ Cemented backfill | - |

Notes: information from the platform of Mining Data Online (<https://miningdataonline.com/>).

**Table S2.** Results of PERMANOVA showing the relative importance of forest type, mining stage and their interaction in structure the phyllosphere communities based on Bray-Curtis dissimilarity.

|  | Df | SumsOfSqs | MeanSqs | F.Model | R^2^ (%) | Pr(>F) |
| --- | --- | --- | --- | --- | --- | --- |
| Bacterial community | | | | | | |
| Mining stage (Stage) | 2 | 2.32 | 1.16 | 4.57 | 4.43 | < 0.001 |
| Forest type (Forest) | 3 | 3.04 | 1.01 | 3.98 | 5.83 | < 0.001 |
| Forest: Stage | 6 | 1.56 | 0.26 | 1.02 | 2.72 | 0.70 |
| Residuals | 198 | 50.31 | 0.25 | 0.88 |  |  |
| Total | 209 | 57.22 | 1.00 |  |  |  |
| Fungal community | | | | | | |
| Mining stage (Stage) | 2 | 2.39 | 1.20 | 3.38 | 2.62 | < 0.001 |
| Forest type (Forest) | 3 | 4.10 | 1.37 | 3.86 | 4.63 | < 0.001 |
| Forest: Stage | 6 | 2.41 | 0.40 | 1.13 | 3.06 | 0.16 |
| Residuals | 197 | 69.77 | 0.35 | 0.89 |  |  |
| Total | 208 ^1^ | 78.67 | 1.00 |  |  |  |

Notes: 1, one outlier was removed from analysis.

**Table S3.** Results of pairwise PERMANOVA for the community composition between mining stages with Benjamini-Hochberg adjustment.

| Pairwise PERMANOVA | Bacterial community | | | Fungal community | | |
| --- | --- | --- | --- | --- | --- | --- |
|  | *F* | R^2^(%) | *P.*adj | *F* | R^2^(%) | *P.*adj |
| Operating vs. Non-operating | 5.18 | 2.99 | **0.001** | 2.34 | 1.38 | **0.001** |
| Operating vs. Controls | 4.50 | 3.35 | **0.001** | 3.27 | 2.47 | **0.001** |
| Non-operating vs. Controls | 4.38 | 3.64 | **0.001** | 3.24 | 2.72 | **0.001** |

**Table S4** Results of pairwise PERMANOVA for the community composition between forest types with Benjamini-Hochberg adjustment.

| Pairwise Permanova | Bacterial community | | | Fungal community | | |
| --- | --- | --- | --- | --- | --- | --- |
|  | *F* | R^2^(%) | *P.*adj | *F* | R^2^(%) | *P.*adj |
| Coniferous vs. Open Canopy | 4.48 | 2.92 | **0.002** | 4.30 | 2.80 | **0.002** |
| Coniferous vs. Mixed | 2.51 | 1.97 | **0.005** | 1.84 | 1.45 | **0.007** |
| Coniferous vs. Deciduous | 5.10 | 3.72 | **0.002** | 3.60 | 2.67 | **0.002** |
| Deciduous vs. Open Canopy | 6.10 | 7.00 | **0.002** | 3.72 | 4.44 | **0.002** |
| Deciduous vs. Mixed | 1.78 | 3.03 | **0.019** | 1.95 | 3.37 | **0.004** |
| Mixed vs. Open Canopy | 4.74 | 6.03 | **0.002** | 4.14 | 5.30 | **0.002** |

**Table S5** Axis loadings of top 15 phyla on first two NMDS axes.

| Bacterial community | | | | | | Fungal community | | | | |
| --- | --- | --- | --- | --- | --- | --- | --- | --- | --- | --- |
| Phylum | NMDS1 | | NMDS2 | R^2^(%) | P | Phylum | NMDS1 | NMDS2 | R^2^(%) | P |
| Acidobacteriota | | -0.4536 | 0.8912 | 71.06 | **0.001** | Aphelidiomycota | 0.8752 | 0.4837 | 22.28 | **0.001** |
| Actinobacteriota | | 0.2105 | -0.9776 | 29.77 | **0.001** | Ascomycota | -0.8799 | 0.4751 | 3.19 | **0.04** |
| Armatimonadota | | 0.0798 | -0.9968 | 16.89 | **0.001** | Basidiobolomycota | -0.8456 | -0.5338 | 0.11 | 0.898 |
| Bacteroidota | | 0.8417 | -0.5400 | 75.6 | **0.001** | Basidiomycota | -0.4225 | -0.9064 | 5.26 | **0.006** |
| Bdellovibrionota | | -0.5691 | 0.8223 | 0.52 | 0.58 | Blastocladiomycota | 0.3326 | -0.9431 | 0.42 | 0.628 |
| Chloroflexi | | 0.9558 | 0.2939 | 60.06 | **0.001** | Chytridiomycota | -0.8925 | -0.4510 | 3.85 | **0.02** |
| Cyanobacteria | | -0.9491 | 0.3150 | 6.89 | **0.007** | Entorrhizomycota | 0.8799 | 0.4752 | 28.2 | **0.001** |
| Desulfobacterota | | 0.9170 | 0.3989 | 69.71 | **0.001** | Glomeromycota | 0.8225 | 0.5688 | 41.67 | **0.001** |
| Firmicutes | | 0.8668 | 0.4986 | 71.24 | **0.001** | Monoblepharomycota | 0.9252 | 0.3795 | 1.31 | 0.226 |
| Gemmatimonadota | | 0.9758 | 0.2185 | 58.73 | **0.001** | Mortierellomycota | 0.8314 | 0.5557 | 51.92 | **0.001** |
| Myxococcota | | 0.6335 | -0.7737 | 9.2 | **0.001** | Mucoromycota | 0.9007 | 0.4345 | 14.83 | **0.001** |
| Planctomycetota | | 0.6962 | 0.7179 | 69.97 | **0.001** | Olpidiomycota | 0.1350 | -0.9909 | 6.98 | **0.002** |
| Proteobacteria | | -0.7515 | -0.6598 | 61.83 | **0.001** | Rozellomycota | 0.9166 | -0.3998 | 1.15 | 0.254 |
| Verrucomicrobiota | | 0.8438 | 0.5367 | 68.61 | **0.001** |  |  |  |  |  |
| WPS-2 | | -0.4839 | 0.8751 | 45.14 | **0.001** |  |  |  |  |  |

**Table S6.** List of bacterial indicators for mining stages with their taxonomic information (only significant species are shown, *P* < 0.05 and stat ≥ 0.2). “Stat” is the association statistic (the square root of the indicator value) and “P value”is the p-value of the permutational test.

| **ASV** | **stat** | **P value** | **Kingdom** | **Phylum** | **Class** | **Order** | **Family** | **Genus** |
| --- | --- | --- | --- | --- | --- | --- | --- | --- |
| **Operating** | |  |  |  |  |  |  |  |
| ASV_11 | 0.29 | 0.002 | Bacteria | Bacteroidota | Bacteroidia | Chitinophagales | Chitinophagaceae | *NA* |
| ASV_19 | 0.276 | 0.002 | Bacteria | Bacteroidota | Bacteroidia | Cytophagales | Microscillaceae | *OLB12* |
| ASV_84 | 0.318 | 0.001 | Bacteria | Proteobacteria | Alphaproteobacteria | Acetobacterales | Acetobacteraceae | *Acidiphilium* |
| ASV_101 | 0.318 | 0.001 | Bacteria | Acidobacteriota | Acidobacteriae | Solibacterales | Solibacteraceae | *Candidatus Solibacter* |
| ASV_159 | 0.317 | 0.001 | Bacteria | Proteobacteria | Alphaproteobacteria | Rhizobiales | Beijerinckiaceae | *Methylobacterium-Methylorubrum* |
| ASV_119 | 0.297 | 0.002 | Bacteria | Desulfobacterota | Desulfuromonadia | Geobacterales | Geobacteraceae | *Geobacter* |
| ASV_91 | 0.29 | 0.003 | Bacteria | Proteobacteria | Alphaproteobacteria | Rhizobiales | Beijerinckiaceae | *1174-901-12* |
| ASV_145 | 0.287 | 0.002 | Bacteria | Proteobacteria | Alphaproteobacteria | Sphingomonadales | Sphingomonadaceae | *Sphingomonas* |
| ASV_326 | 0.284 | 0.001 | Bacteria | Actinobacteriota | Actinobacteria | Frankiales | Nakamurellaceae | *Nakamurella* |
| ASV_49 | 0.276 | 0.004 | Bacteria | Acidobacteriota | Acidobacteriae | Acidobacteriales | Acidobacteriaceae | *Occallatibacter* |
| ASV_178 | 0.268 | 0.001 | Bacteria | Proteobacteria | Alphaproteobacteria | Caulobacterales | Hyphomonadaceae | *SWB02* |
| ASV_156 | 0.267 | 0.003 | Bacteria | Proteobacteria | Alphaproteobacteria | Rhizobiales | Xanthobacteraceae | *Pseudorhodoplanes* |
| ASV_360 | 0.266 | 0.004 | Bacteria | Proteobacteria | Alphaproteobacteria | Acetobacterales | Acetobacteraceae | *Acidiphilium* |
| ASV_80 | 0.26 | 0.003 | Bacteria | Actinobacteriota | Thermoleophilia | Solirubrobacterales | 67-14 | *NA* |
| ASV_721 | 0.258 | 0.001 | Bacteria | Acidobacteriota | Acidobacteriae | Acidobacteriales | Acidobacteriaceae | *Bryocella* |
| ASV_160 | 0.251 | 0.004 | Bacteria | Proteobacteria | Alphaproteobacteria | Sphingomonadales | Sphingomonadaceae | *Sphingomonas* |
| ASV_385 | 0.249 | 0.001 | Bacteria | Bacteroidota | Bacteroidia | Chitinophagales | Chitinophagaceae | *Aurantisolimonas* |
| ASV_150 | 0.246 | 0.005 | Bacteria | Proteobacteria | Gammaproteobacteria | Burkholderiales | Nitrosomonadaceae | *IS-44* |
| ASV_507 | 0.246 | 0.001 | Bacteria | Actinobacteriota | Actinobacteria | Micromonosporales | Micromonosporaceae | *Actinoplanes* |
| ASV_161 | 0.245 | 0.001 | Bacteria | Proteobacteria | Gammaproteobacteria | Burkholderiales | Oxalobacteraceae | *Massilia* |
| ASV_187 | 0.244 | 0.006 | Bacteria | Firmicutes | Desulfitobacteriia | Desulfitobacteriales | Desulfitobacteriaceae | *Desulfosporosinus* |
| ASV_214 | 0.244 | 0.005 | Bacteria | Bacteroidota | SJA-28 | NA | NA | *NA* |
| ASV_580 | 0.244 | 0.001 | Bacteria | Bacteroidota | Bacteroidia | Cytophagales | Spirosomaceae | *Huanghella* |
| ASV_472 | 0.239 | 0.001 | Bacteria | Bacteroidota | Bacteroidia | Cytophagales | Spirosomaceae | *Spirosoma* |
| ASV_57 | 0.238 | 0.006 | Bacteria | Verrucomicrobiota | Verrucomicrobiae | Opitutales | Opitutaceae | *Lacunisphaera* |
| ASV_207 | 0.238 | 0.005 | Bacteria | Proteobacteria | Gammaproteobacteria | Burkholderiales | Comamonadaceae | *NA* |
| ASV_332 | 0.23 | 0.001 | Bacteria | Proteobacteria | Alphaproteobacteria | Rhizobiales | Beijerinckiaceae | *1174-901-12* |
| ASV_131 | 0.226 | 0.011 | Bacteria | Acidobacteriota | Acidobacteriae | Bryobacterales | Bryobacteraceae | *Bryobacter* |
| ASV_333 | 0.226 | 0.006 | Bacteria | Bacteroidota | Bacteroidia | Chitinophagales | Chitinophagaceae | *Ferruginibacter* |
| ASV_379 | 0.221 | 0.003 | Bacteria | Proteobacteria | Alphaproteobacteria | Sphingomonadales | Sphingomonadaceae | *Sphingomonas* |
| ASV_733 | 0.218 | 0.002 | Bacteria | Deinococcota | Deinococci | Deinococcales | Deinococcaceae | *Deinococcus* |
| ASV_508 | 0.217 | 0.004 | Bacteria | Proteobacteria | Alphaproteobacteria | Rhizobiales | Beijerinckiaceae | *1174-901-12* |
| ASV_1065 | 0.215 | 0.007 | Bacteria | Proteobacteria | Alphaproteobacteria | Acetobacterales | Acetobacteraceae | *Acidiphilium* |
| ASV_188 | 0.209 | 0.002 | Bacteria | Proteobacteria | Alphaproteobacteria | Acetobacterales | Acetobacteraceae | *Acidiphilium* |
| ASV_27 | 0.204 | 0.017 | Bacteria | Proteobacteria | Alphaproteobacteria | Sphingomonadales | Sphingomonadaceae | *Sphingomonas* |
| **non-operating** | |  |  |  |  |  |  |  |
| ASV_8 | 0.279 | 0.001 | Bacteria | Proteobacteria | Alphaproteobacteria | Acetobacterales | Acetobacteraceae | *NA* |
| ASV_34 | 0.275 | 0.001 | Bacteria | Proteobacteria | Alphaproteobacteria | Caulobacterales | Caulobacteraceae | *PMMR1* |
| ASV_147 | 0.266 | 0.001 | Bacteria | Proteobacteria | Alphaproteobacteria | Acetobacterales | Acetobacteraceae | *NA* |
| ASV_208 | 0.266 | 0.001 | Bacteria | Acidobacteriota | Acidobacteriae | Acidobacteriales | Acidobacteriaceae | *Granulicella* |
| ASV_132 | 0.262 | 0.001 | Bacteria | Proteobacteria | Alphaproteobacteria | Sphingomonadales | Sphingomonadaceae | *Novosphingobium* |
| ASV_146 | 0.26 | 0.003 | Bacteria | Acidobacteriota | Acidobacteriae | Acidobacteriales | Acidobacteriaceae | *Bryocella* |
| ASV_116 | 0.25 | 0.003 | Bacteria | Proteobacteria | Alphaproteobacteria | Acetobacterales | Acetobacteraceae | *NA* |
| ASV_194 | 0.245 | 0.004 | Bacteria | Proteobacteria | Alphaproteobacteria | Acetobacterales | Acetobacteraceae | *NA* |
| ASV_30 | 0.243 | 0.004 | Bacteria | Proteobacteria | Alphaproteobacteria | Acetobacterales | Acetobacteraceae | *NA* |
| ASV_227 | 0.241 | 0.001 | Bacteria | Acidobacteriota | Acidobacteriae | Acidobacteriales | Acidobacteriaceae | *Granulicella* |
| ASV_88 | 0.233 | 0.006 | Bacteria | Proteobacteria | Alphaproteobacteria | Acetobacterales | Acetobacteraceae | *NA* |
| ASV_169 | 0.231 | 0.004 | Bacteria | Proteobacteria | Alphaproteobacteria | Sphingomonadales | Sphingomonadaceae | *Novosphingobium* |
| ASV_85 | 0.23 | 0.004 | Bacteria | Bacteroidota | Bacteroidia | Chitinophagales | Chitinophagaceae | *Aurantisolimonas* |
| ASV_66 | 0.228 | 0.01 | Bacteria | Acidobacteriota | Acidobacteriae | Acidobacteriales | Acidobacteriaceae | *Granulicella* |
| ASV_250 | 0.222 | 0.001 | Bacteria | Proteobacteria | Alphaproteobacteria | Caulobacterales | Caulobacteraceae | *NA* |
| ASV_86 | 0.221 | 0.011 | Bacteria | Acidobacteriota | Acidobacteriae | Acidobacteriales | Acidobacteriaceae | *Granulicella* |
| ASV_112 | 0.221 | 0.011 | Bacteria | Proteobacteria | Alphaproteobacteria | Acetobacterales | Acetobacteraceae | *Acidisoma* |
| ASV_89 | 0.217 | 0.007 | Bacteria | Proteobacteria | Alphaproteobacteria | Caulobacterales | Caulobacteraceae | *NA* |
| ASV_94 | 0.214 | 0.007 | Bacteria | Acidobacteriota | Acidobacteriae | Acidobacteriales | Acidobacteriaceae | *Bryocella* |
| ASV_51 | 0.213 | 0.005 | Bacteria | Proteobacteria | Alphaproteobacteria | Acetobacterales | Acetobacteraceae | *Acidiphilium* |
| ASV_100 | 0.213 | 0.01 | Bacteria | Planctomycetota | Planctomycetes | Isosphaerales | Isosphaeraceae | *Tundrisphaera* |
| ASV_74 | 0.209 | 0.009 | Bacteria | Proteobacteria | Alphaproteobacteria | Acetobacterales | Acetobacteraceae | *Acidisoma* |
| ASV_354 | 0.204 | 0.013 | Bacteria | Acidobacteriota | Acidobacteriae | Acidobacteriales | Acidobacteriaceae | *Granulicella* |
| ASV_148 | 0.203 | 0.022 | Bacteria | Proteobacteria | Alphaproteobacteria | Acetobacterales | Acetobacteraceae | *Acidisphaera* |
| ASV_192 | 0.203 | 0.015 | Bacteria | Proteobacteria | Alphaproteobacteria | Acetobacterales | Acetobacteraceae | *NA* |
| **Controls** |  |  |  |  |  |  |  |  |
| ASV_361 | 0.514 | 0.001 | Bacteria | Proteobacteria | Gammaproteobacteria | Burkholderiales | Burkholderiaceae | *Cupriavidus* |
| ASV_543 | 0.386 | 0.001 | Bacteria | Acidobacteriota | Acidobacteriae | Subgroup 2 | NA | *NA* |
| ASV_373 | 0.364 | 0.001 | Bacteria | Proteobacteria | Alphaproteobacteria | Rhizobiales | Beijerinckiaceae | *Roseiarcus* |
| ASV_244 | 0.358 | 0.001 | Bacteria | Proteobacteria | Gammaproteobacteria | Salinisphaerales | Solimonadaceae | *Nevskia* |
| ASV_278 | 0.358 | 0.001 | Bacteria | Proteobacteria | Gammaproteobacteria | Xanthomonadales | Xanthomonadaceae | *Stenotrophomonas* |
| ASV_245 | 0.347 | 0.001 | Bacteria | Bacteroidota | Bacteroidia | Chitinophagales | Chitinophagaceae | *Puia* |
| ASV_299 | 0.307 | 0.001 | Bacteria | Proteobacteria | Gammaproteobacteria | Xanthomonadales | Rhodanobacteraceae | *Rhodanobacter* |
| ASV_38 | 0.299 | 0.001 | Bacteria | Proteobacteria | Alphaproteobacteria | Rhizobiales | Xanthobacteraceae | *Bradyrhizobium* |
| ASV_199 | 0.299 | 0.001 | Bacteria | Proteobacteria | Gammaproteobacteria | WD260 | NA | *NA* |
| ASV_226 | 0.258 | 0.001 | Bacteria | Proteobacteria | Gammaproteobacteria | Burkholderiales | Comamonadaceae | *Variovorax* |
| ASV_168 | 0.241 | 0.001 | Bacteria | Proteobacteria | Gammaproteobacteria | Pseudomonadales | Pseudomonadaceae | *Pseudomonas* |
| ASV_167 | 0.212 | 0.006 | Bacteria | Acidobacteriota | Acidobacteriae | Acidobacteriales | Acidobacteriaceae | *Granulicella* |
| ASV_106 | 0.208 | 0.01 | Bacteria | Bacteroidota | Bacteroidia | Sphingobacteriales | Sphingobacteriaceae | *Mucilaginibacter* |

**Table S7.** List of fungal indicators for mining stages with their taxonomic information (only significant species are shown P < 0.05 and stat ≥ 0.2). “Stat” is the association statistic (the square root of the indicator value) and “P value”is the p-value of the permutational test.

| ASV | stat | P value | | Kingdom | Phylum | Class | Order | Family | Genus |
| --- | --- | --- | --- | --- | --- | --- | --- | --- | --- |
| **Operating** | | |  |  |  |  |  |  |  |
| ASV_10 | 0.326 | | 0.001 | Fungi | Basidiomycota | Microbotryomycetes | Kriegeriales | Kriegeriaceae | *Phenoliferia* |
| ASV_21 | 0.32 | | 0.001 | Fungi | Chytridiomycota | Spizellomycetes | Spizellomycetales | NA | NA |
| ASV_86 | 0.32 | | 0.001 | Fungi | Chytridiomycota | Spizellomycetes | Spizellomycetales | Powellomycetaceae | *Thoreauomyces* |
| ASV_115 | 0.314 | | 0.001 | Fungi | Ascomycota | Dothideomycetes | Mytilinidiales | Mytilinidiaceae | *Lophium* |
| ASV_212 | 0.259 | | 0.001 | Fungi | Ascomycota | Dothideomycetes | Mytilinidiales | Mytilinidiaceae | *Lophium* |
| ASV_98 | 0.256 | | 0.001 | Fungi | Basidiomycota | Tremellomycetes | Tremellales | NA | NA |
| ASV_235 | 0.254 | | 0.001 | Fungi | Basidiomycota | Microbotryomycetes | Sporidiobolales | Sporidiobolaceae | *Rhodosporidiobolus* |
| ASV_453 | 0.253 | | 0.002 | Fungi | Ascomycota | Dothideomycetes | Capnodiales | Teratosphaeriaceae | *Devriesia* |
| ASV_29 | 0.247 | | 0.003 | Fungi | Chytridiomycota | Rhizophydiomycetes | Rhizophydiales | Rhizophydiaceae | *Rhizophydium* |
| ASV_193 | 0.245 | | 0.004 | Fungi | Chytridiomycota | Rhizophydiomycetes | Rhizophydiales | Rhizophydiaceae | *Rhizophydium* |
| ASV_314 | 0.245 | | 0.001 | Fungi | Ascomycota | Dothideomycetes | Pleosporales | Phaeosphaeriaceae | *Setomelanomma* |
| ASV_250 | 0.241 | | 0.004 | Fungi | NA | NA | NA | NA | NA |
| ASV_616 | 0.24 | | 0.004 | Fungi | Ascomycota | Dothideomycetes | Dothideales | Dothideaceae | *Rhizosphaera* |
| ASV_414 | 0.237 | | 0.003 | Fungi | NA | NA | NA | NA | NA |
| ASV_127 | 0.233 | | 0.004 | Fungi | Basidiomycota | Tremellomycetes | Cystofilobasidiales | Mrakiaceae | *Mrakia* |
| ASV_59 | 0.228 | | 0.002 | Fungi | Ascomycota | NA | NA | NA | NA |
| ASV_100 | 0.226 | | 0.01 | Fungi | Ascomycota | Dothideomycetes | Capnodiales | Neodevriesiaceae | NA |
| ASV_217 | 0.223 | | 0.005 | Fungi | NA | NA | NA | NA | NA |
| ASV_39 | 0.221 | | 0.011 | Fungi | Ascomycota | Leotiomycetes | Leotiales | Mniaeciaceae | *Epithamnolia* |
| ASV_199 | 0.219 | | 0.004 | Fungi | Chytridiomycota | Spizellomycetes | Spizellomycetales | NA | NA |
| ASV_698 | 0.219 | | 0.006 | Fungi | NA | NA | NA | NA | NA |
| ASV_169 | 0.218 | | 0.009 | Fungi | Ascomycota | Dothideomycetes | Capnodiales | Teratosphaeriaceae | *Capnobotryella* |
| ASV_897 | 0.218 | | 0.012 | Fungi | Basidiomycota | Tremellomycetes | Tremellales | NA | NA |
| ASV_75 | 0.217 | | 0.006 | Fungi | Ascomycota | Pezizomycotina_  cls_Incertae_sedis | Pezizomycotina_  ord_Incertae_sedis | Pezizomycotina_  fam_Incertae_sedis | *Ciliophora* |
| ASV_6 | 0.217 | | 0.013 | Fungi | NA | NA | NA | NA | NA |
| ASV_117 | 0.214 | | 0.003 | Fungi | NA | NA | NA | NA | NA |
| ASV_164 | 0.209 | | 0.005 | Fungi | Ascomycota | Dothideomycetes | Venturiales | Venturiaceae | *Venturia* |
| ASV_622 | 0.208 | | 0.003 | Fungi | Basidiomycota | Tremellomycetes | Filobasidiales | Piskurozymaceae | NA |
| ASV_1240 | 0.207 | | 0.007 | Fungi | NA | NA | NA | NA | NA |
| ASV_352 | 0.204 | | 0.007 | Fungi | NA | NA | NA | NA | NA |
| ASV_544 | 0.204 | | 0.011 | Fungi | Basidiomycota | Agaricomycetes | Cantharellales | Ceratobasidiaceae | *Ceratobasidium* |
| ASV_316 | 0.2 | | 0.012 | Fungi | Olpidiomycota | GS18 | NA | NA | NA |
| **Non-operating** | | |  |  |  |  |  |  |  |
| ASV_136 | 0.314 | | 0.001 | Fungi | Ascomycota | Orbiliomycetes | Orbiliales | NA | NA |
| ASV_186 | 0.245 | | 0.004 | Fungi | Ascomycota | Eurotiomycetes | Chaetothyriales | NA | NA |
| ASV_50 | 0.222 | | 0.005 | Fungi | Ascomycota | Dothideomycetes | Dothideales | NA | NA |
| ASV_244 | 0.222 | | 0.001 | Fungi | Ascomycota | Eurotiomycetes | Chaetothyriales | Herpotrichiellaceae | *Cladophialophora* |
| ASV_155 | 0.22 | | 0.005 | Fungi | Ascomycota | Eurotiomycetes | Chaetothyriales | Herpotrichiellaceae | NA |
| ASV_504 | 0.212 | | 0.009 | Fungi | Ascomycota | Eurotiomycetes | Chaetothyriales | Herpotrichiellaceae | *Cladophialophora* |
| ASV_188 | 0.208 | | 0.01 | Fungi | Ascomycota | Leotiomycetes | Helotiales | NA | NA |
| **Controls** |  | |  |  |  |  |  |  |  |
| ASV_429 | 0.397 | | 0.001 | Fungi | Basidiomycota | Agaricomycetes | Thelephorales | Thelephoraceae | *Thelephora* |
| ASV_634 | 0.395 | | 0.001 | Fungi | Ascomycota | Pezizomycotina_  cls_Incertae_sedis | Pezizomycotina_  ord_Incertae_sedis | Pezizomycotina_  fam_Incertae_sedis | *Ciliophora* |
| ASV_729 | 0.395 | | 0.001 | Fungi | NA | NA | NA | NA | *NA* |
| ASV_659 | 0.391 | | 0.001 | Fungi | Ascomycota | Pezizomycotina_  cls_Incertae_sedis | Pezizomycotina_  ord_Incertae_sedis | Pezizomycotina_  fam_Incertae_sedis | *Ciliophora* |
| ASV_502 | 0.389 | | 0.001 | Fungi | Basidiomycota | Tremellomycetes | Filobasidiales | Piskurozymaceae | *Solicoccozyma* |
| ASV_673 | 0.388 | | 0.001 | Fungi | Ascomycota | Leotiomycetes | Helotiales | NA | *NA* |
| ASV_675 | 0.386 | | 0.001 | Fungi | Ascomycota | Eurotiomycetes | Eurotiales | Trichocomaceae | *Talaromyces* |
| ASV_441 | 0.384 | | 0.001 | Fungi | Basidiomycota | Agaricomycetes | Agaricales | Inocybaceae | *Inocybe* |
| ASV_490 | 0.384 | | 0.001 | Fungi | Ascomycota | Dothideomycetes | Pleosporales | Phaeosphaeriaceae | *Paraphoma* |
| ASV_542 | 0.384 | | 0.001 | Fungi | Mucoromycota | Umbelopsidomycetes | Umbelopsidales | Umbelopsidaceae | *Umbelopsis* |
| ASV_509 | 0.383 | | 0.001 | Fungi | NA | NA | NA | NA | *NA* |
| ASV_603 | 0.383 | | 0.001 | Fungi | Basidiomycota | Agaricomycetes | Russulales | Russulaceae | *Lactifluus* |
| ASV_559 | 0.382 | | 0.001 | Fungi | Ascomycota | Leotiomycetes | Helotiales | Hyaloscyphaceae | *NA* |
| ASV_405 | 0.376 | | 0.001 | Fungi | Ascomycota | Leotiomycetes | Helotiales | Helotiaceae | *Ascocoryne* |
| ASV_76 | 0.373 | | 0.001 | Fungi | Ascomycota | Pezizomycetes | Pezizales | Pyronemataceae | *Sphaerosporella* |
| ASV_389 | 0.373 | | 0.001 | Fungi | Ascomycota | Leotiomycetes | Helotiales | NA | *NA* |
| ASV_266 | 0.372 | | 0.001 | Fungi | Ascomycota | Leotiomycetes | Helotiales | Helotiales_fam_  Incertae_sedis | *Leptodontidium* |
| ASV_123 | 0.371 | | 0.001 | Fungi | NA | NA | NA | NA | *NA* |
| ASV_355 | 0.371 | | 0.001 | Fungi | NA | NA | NA | NA | *NA* |
| ASV_379 | 0.371 | | 0.001 | Fungi | NA | NA | NA | NA | *NA* |
| ASV_19 | 0.37 | | 0.001 | Fungi | NA | NA | NA | NA | *NA* |
| ASV_454 | 0.369 | | 0.001 | Fungi | Ascomycota | Leotiomycetes | Helotiales | Hyaloscyphaceae | *Hyaloscypha* |
| ASV_159 | 0.368 | | 0.001 | Fungi | Ascomycota | Leotiomycetes | Helotiales | Helotiaceae | *Meliniomyces* |
| ASV_61 | 0.365 | | 0.001 | Fungi | Basidiomycota | Microbotryomycetes | Leucosporidiales | Leucosporidiaceae | *Leucosporidium* |
| ASV_738 | 0.364 | | 0.001 | Fungi | Ascomycota | Leotiomycetes | Thelebolales | Pseudeurotiaceae | *NA* |
| ASV_101 | 0.363 | | 0.001 | Fungi | Basidiomycota | Agaricomycetes | Agaricales | Strophariaceae | *Hypholoma* |
| ASV_176 | 0.363 | | 0.001 | Fungi | Basidiomycota | Agaricomycetes | Agaricales | Tricholomataceae | *Mycena* |
| ASV_411 | 0.362 | | 0.001 | Fungi | Ascomycota | Leotiomycetes | Thelebolales | NA | *NA* |
| ASV_182 | 0.359 | | 0.001 | Fungi | NA | NA | NA | NA | *NA* |
| ASV_530 | 0.359 | | 0.001 | Fungi | Ascomycota | Eurotiomycetes | Chaetothyriales | Herpotrichiellaceae | *NA* |
| ASV_589 | 0.358 | | 0.001 | Fungi | NA | NA | NA | NA | *NA* |
| ASV_228 | 0.354 | | 0.001 | Fungi | NA | NA | NA | NA | *NA* |
| ASV_281 | 0.353 | | 0.001 | Fungi | NA | NA | NA | NA | *NA* |
| ASV_118 | 0.352 | | 0.001 | Fungi | Ascomycota | Sordariomycetes | Xylariales | Xylariaceae | *Hypoxylon* |
| ASV_214 | 0.351 | | 0.001 | Fungi | Ascomycota | Sordariomycetes | Xylariales | Xylariaceae | *Hypoxylon* |
| ASV_309 | 0.349 | | 0.001 | Fungi | Basidiomycota | Microbotryomycetes | Kriegeriales | Kriegeriaceae | *Phenoliferia* |
| ASV_242 | 0.347 | | 0.001 | Fungi | Mortierellomycota | Mortierellomycetes | Mortierellales | Mortierellaceae | *Mortierella* |
| ASV_631 | 0.345 | | 0.001 | Fungi | Ascomycota | Saccharomycetes | Saccharomycetales | Saccharomycetales_  fam_Incertae_sedis | *Nadsonia* |
| ASV_567 | 0.333 | | 0.001 | Fungi | Basidiomycota | Agaricomycetes | Agaricales | Hymenogastraceae | *Hebeloma* |
| ASV_187 | 0.296 | | 0.001 | Fungi | Ascomycota | Leotiomycetes | Helotiales | Hyaloscyphaceae | *Hyaloscypha* |
| ASV_398 | 0.287 | | 0.001 | Fungi | Ascomycota | Leotiomycetes | Helotiales | Helotiales_fam_  Incertae_sedis | *Leptodontidium* |
| ASV_525 | 0.273 | | 0.001 | Fungi | Ascomycota | Eurotiomycetes | Chaetothyriales | NA | *NA* |
| ASV_143 | 0.259 | | 0.001 | Fungi | Ascomycota | Leotiomycetes | Helotiales | Helotiaceae | *Collophora* |
| ASV_344 | 0.256 | | 0.001 | Fungi | Ascomycota | Leotiomycetes | Helotiales | Helotiales_fam_  Incertae_sedis | *Cadophora* |
| ASV_395 | 0.252 | | 0.003 | Fungi | Ascomycota | Eurotiomycetes | Chaetothyriales | Herpotrichiellaceae | *Rhinocladiella* |
| ASV_333 | 0.246 | | 0.001 | Fungi | Ascomycota | NA | NA | NA | *NA* |
| ASV_442 | 0.244 | | 0.005 | Fungi | Ascomycota | Dothideomycetes | Capnodiales | NA | *NA* |
| ASV_110 | 0.241 | | 0.005 | Fungi | Ascomycota | Eurotiomycetes | Chaetothyriales | NA | *NA* |
| ASV_318 | 0.239 | | 0.003 | Fungi | Basidiomycota | Agaricomycetes | Thelephorales | Thelephoraceae | *Thelephora* |
| ASV_678 | 0.232 | | 0.002 | Fungi | Ascomycota | Dothideomycetes | Venturiales | Venturiaceae | *Venturia* |
| ASV_321 | 0.231 | | 0.002 | Fungi | Ascomycota | NA | NA | NA | *NA* |
| ASV_608 | 0.229 | | 0.001 | Fungi | NA | NA | NA | NA | *NA* |
| ASV_606 | 0.22 | | 0.007 | Fungi | Basidiomycota | Tremellomycetes | Cystofilobasidiales | Mrakiaceae | *Mrakia* |
| ASV_239 | 0.218 | | 0.006 | Fungi | Basidiomycota | Agaricomycetes | Polyporales | Ganodermataceae | *Ganoderma* |
| ASV_356 | 0.207 | | 0.013 | Fungi | Ascomycota | Leotiomycetes | Helotiales | Leotiaceae | *Pezoloma* |
| ASV_285 | 0.202 | | 0.01 | Fungi | Ascomycota | Eurotiomycetes | Chaetothyriales | NA | *NA* |
| ASV_78 | 0.2 | | 0.012 | Fungi | Chytridiomycota | Spizellomycetes | Spizellomycetales | NA | *NA* |

**Table S8.**  Phyllosphere alpha diversity at each distance level from mine sites. Mean ± SD are shown. Significant p-values for the effects of the distance, mining stage and their interactions were calculated with ANOVA using Type II sum of squares based on the results of GLMMs. Different lowercase letters represent significant multiple compositions with Tukey’s post hoc tests at the 0.05 level. Significant relationships are highlighted in bold.

| Alpha diversity | **ANOVA** (type II sums) | | **Distance from mine sites (km)** | | | |
| --- | --- | --- | --- | --- | --- | --- |
|  | Distance | Distance* Stage | 0 (N=39) | 0.05 (N=44) | 0.2 (N=43) | 1 (N=44) |
| **Bacteria** |  |  |  |  |  |  |
| Observed | **p<0.01** | p=0.63 | 640.85 ± 180.97 b | 584.09 ± 180.41 ab | 550.28 ± 183.82 a | 568.25 ± 190.34 a |
| Shannon | **p<0.01** | p=0.86 | 5.72 ± 0.43 b | 5.55 ± 0.47 ab | 5.45 ± 0.50 a | 5.51 ± 0.43 a |
| InvSimpson | **p=0.03** | p=0.81 | 153.42 ± 78.92 a | 127.28 ± 68.16 a | 115.77 ± 59.95 a | 119.45 ± 63.43 a |
| **Fungi** |  |  |  |  |  |  |
| Observed | **p<0.01** | p=0.47 | 462.50 ± 142.32 b | 382.16 ± 104.14 a | 396.40 ± 129.17 a | 404.80 ± 113.62 ab |
| Shannon | **p=0.01** | p=0.64 | 4.88 ± 0.38 b | 4.62 ± 0.47 a | 4.69 ± 0.41 ab | 4.69 ± 0.48 ab |
| InvSimpson | p=0.33 | p=0.42 | 55.31 ± 21.65 a | 45.84 ± 25.13 a | 47.96 ± 22.99 a | 48.34 ± 25.71 a |

**Table S9.** PERMANOVA results showing the relative importance of distance from mines, mining stage and their interactions in structing the phyllosphere microbial communities based on Bray-Curtis dissimilarity.

|  | Df | SumsOfSqs | MeanSqs | F.Model | R^2^ (%) | Pr(>F) |
| --- | --- | --- | --- | --- | --- | --- |
| **Bacterial community** | | | | | | |
| Mining stage (Stage) | 1 | 1.34 | 1.34 | 5.09 | 3% | **< 0.001** |
| Distance | 1 | 0.66 | 0.66 | 2.50 | 1% | **< 0.001** |
| Distance: Stage | 1 | 0.33 | 0.33 | 1.26 | 1% | 0.07 |
| Residuals | 166 | 43.58 | 0.26 | 0.95 |  |  |
| Total | 169 | 45.91 | 1.00 |  |  |  |
| **Fungal community** | | | | | | |
| Mining stage (Stage) | 1 | 0.97 | 0.97 | 2.67 | 2% | **< 0.001** |
| Distance | 1 | 0.64 | 0.64 | 1.74 | 1% | **< 0.001** |
| Distance: Stage | 1 | 0.42 | 0.42 | 1.14 | 1% | 0.06 |
| Residuals | 165 | 60.17 | 0.36 | 0.97 |  |  |
| Total | 168 | 62.20 | 1.00 |  |  |  |

**Table S****10.** Relative abundance of main phyllophere phyla (i.e. relative abundance > 0.5 through all samples) at each distance from mine sites. Mean ± SD are shown. Zero-inflated beta mixed regression models were used to compare mean relative abundance of phyllosphere phylum. Lowercase letters represent significant differences based on Tukey’s test at the 0.05 level. Significant relationships (significant differences in Tukey’s test at 0.05 level) are highlighted in bold.

| **Phylum** | **Mining Stage** | **Distance (km)** | | | |
| --- | --- | --- | --- | --- | --- |
|  |  | **0** | **0.05** | **0.2** | **1** |
| **Bacteria** | | | | | |
| Proteobacteria |  | 52.28 ± 9.7 a | 54.49 ± 8.56 a | 55.43 ± 7.89 a | 55.2 ± 8.01 a |
| **Acidobacteriota *** | Operating | 11.96 ± 5.69 a | 15.28 ± 5.63 ab | 18.07 ± 6.02 b | 19.52 ± 4.8 b |
|  | Non-Operating | 19.82 ± 7.07 a | 18.48 ± 6.53 a | 22.36 ± 6.78 a | 20.13 ± 7.3 a |
| **Bacteroidota *** | Operating | 16.74 ± 5.98 b | 12.68 ± 6.77 a | 10.34 ± 6.06 a | 9.57 ± 6.06 a |
|  | Non-Operating | 10.09 ± 5.52 a | 10.13 ± 5.9 a | 5.68 ± 3.83 a | 7.43 ± 5.16 a |
| **Actinobacteriota *** | Operating | 5.33 ± 2.22 b | 5.22 ± 3.58 ab | 4.29 ± 2.02 ab | 3.82 ± 1.75 a |
|  | Non-Operating | 4.38 ± 1.88 a | 4.39 ± 1.62 a | 3.53 ± 1.45 a | 4.45 ± 2.04 a |
| Planctomycetota |  | 3.45 ± 1.86 a | 2.75 ± 1.65 a | 2.77 ± 1.4 a | 3.14 ± 1.69 a |
| Verrucomicrobiota |  | 2.46 ± 1.71 a | 1.86 ± 1.51 a | 1.84 ± 1.5 a | 2.14 ± 1.61 a |
| Cyanobacteria |  | 0.98 ± 1.06 a | 1.8 ± 1.95 a | 2.33 ± 4.04 a | 1.33 ± 1.46 a |
| Myxococcota |  | 1.59 ± 0.95 a | 1.74 ± 1.82 a | 1.22 ± 0.75 a | 1.4 ± 0.71 a |
| **WPS-2 *** | Operating | 0.17± 0.15 a | 0.40 ± 0.31 ab | 0.77 ± 0.80 b | 1.01 ± 0.88 b |
|  | Non-Operating | 0.89 ± 0.79 a | 0.971 ± 0.92 a | 1.54 ± 1.20 a | 1.13 ± 0.86 a |
| Firmicutes |  | 1.04 ± 1.27 a | 0.72 ± 1.19 a | 0.71 ± 1.16 a | 0.86 ± 1.35 a |
| **Armatimonadota *** | Operating | 0.96 ± 0.6 b | 0.94 ± 0.52 b | 0.81 ± 0.41 ab | 0.61 ± 0.36 a |
|  | Non-Operating | 0.92 ± 0.38 b | 0.56 ± 0.22 a | 0.53 ± 0.35 a | 0.61 ± 0.28 ab |
| **Fungi** | | | | | |
| Ascomycota |  | 56.42 ± 10.23 a | 55.21 ± 12.86 a | 54.72 ± 10.61 a | 54.32 ± 12.08 a |
| Basidiomycota |  | 18.53 ± 9.75 a | 19.84 ± 10.94 a | 21.31 ± 9.72 a | 22.41 ± 10.26 a |
| Chytridiomycota |  | 8.98 ± 7.1 a | 9.02 ± 5.84 a | 9.95 ± 8.6 a | 7.59 ± 6.4 a |
| Olpidiomycota |  | 2.76 ± 2.94 a | 2.61 ± 2.68 a | 2.63 ± 2.87 a | 3.06 ± 4.21 a |

**Table S11.** Relative abundance of top 10 phyllophere genera at each distance from mine sites. Mean ± SD are shown. Zero-inflated beta mixed regression models were used to compare mean relative abundance of phyllosphere genera. Lowercase letters represent significant differences based on Tukey’s test at the 0.05 level. Significant relationships (i.e. significant differences in Tukey test at 0.05 level) are highlighted in bold.

| **Genus** | **Mining Stage** | **Distance (km)** | | | |
| --- | --- | --- | --- | --- | --- |
|  |  | **0** | **0.05** | **0.2** | **1** |
| **Bacterial genus** |  |  |  |  |  |
| ***Granulicella **** | Operating | 6.33 ± 5.3 a | 8.57 ± 4.71 b | 11.07 ± 4.56 bc | 11.77 ± 4.32 c |
|  | Non-Operating | 11.91 ± 5.12 a | 11.94 ± 4.06 a | 13.75 ± 4.08 a | 12.15 ± 4.28 a |
| ***Sphingomonas **** | Operating | 7.03 ± 3.61 b | 5.85 ± 3.39 ab | 5.55 ± 3.32 ab | 4.19 ± 2.63 a |
|  | Non-Operating | 5.61 ± 2.87 a | 6.68 ± 3.74 a | 4.43 ± 2.5 a | 6.3 ± 3.53 a |
| *Acidiphilium* |  | 4.36 ± 2.68 a | 4.17 ± 2.03 a | 4.85 ± 2.92 a | 3.8 ± 1.74 a |
| *g117490112* |  | 3.06 ± 1.71 a | 3.3 ± 1.54 a | 3.44 ± 1.53 a | 3.5 ± 1.39 a |
| *Mucilaginibacter* |  | 2.88 ± 1.48 a | 3.01 ± 1.42 a | 2.64 ± 1.2 a | 2.99 ± 1.6 a |
| *Variovorax* |  | 1.72 ± 2.2 a | 1.84 ± 2.49 a | 2.38 ± 3.71 a | 2.7 ± 4.06 a |
| ***Leptothrix **** | Operating | 1.72 ± 1.22 a | 2.74 ± 1.75 b | 2.68 ± 1.22 b | 3.1 ± 2.47 b |
|  | Non-Operating | 2.63 ± 1.43 a | 2.56 ± 1.32 a | 2.26 ± 1.4 a | 2.41 ± 1.48 a |
| *Rhizobacter* |  | 2.51 ± 1.69 a | 2.37 ± 1.44 a | 2.63 ± 1.46 a | 2.09 ± 1.26 a |
| *Limnobacter* |  | 2.27 ± 1.52 a | 2.37 ± 1.36 a | 2.25 ± 1.14 a | 2.19 ± 1.22 a |
| ***Terriglobus **** | Operating | 1.3 ± 1.03 a | 2.11 ± 2.12 b | 2.06 ± 1.04 b | 1.96 ± 1.1 b |
|  | Non-Operating | 2.23 ± 1.18 a | 2.39 ± 0.96 a | 2.62 ± 1.39 a | 2.47 ± 1.2 a |
| **Fungal genus** |  |  |  |  |  |
| *Phenoliferia* |  | 6.08 ± 5.89 a | 6.67 ± 4.9 a | 7.65 ± 5.89 a | 6.73 ± 5.14 a |
| *Sporormiella* |  | 2.41 ± 3.79 a | 4.19 ± 8.67 a | 3.46 ± 6.64 a | 3.93 ± 8.62 a |
| *Cladophialophora* |  | 2.96 ± 3.03 a | 1.98 ± 1.97 a | 2.16 ± 3.34 a | 2.19 ± 2.47 a |
| *Ciliophora* |  | 2.22 ± 2.26 a | 1.72 ± 1.45 a | 1.63 ± 1.3 a | 1.54 ± 1.43 a |
| *Epibryon* |  | 1.28 ± 1.89 a | 1.41 ± 1.52 a | 1.47 ± 1.43 a | 1 ± 0.89 a |
| ***Epithamnolia **** |  | 0.93 ± 1.15 a | 1.26 ± 1.99 ab | 1.58 ± 1.55 ab | 2.14 ± 1.99 b |
| ***Thoreauomyces **** | Operating | 0.93 ± 1.2 a | 0.98 ± 0.96 a | 1.96 ± 2.14 b | 1.61 ± 1.69 ab |
|  | Non-Operating | 1.25 ± 0.69 a | 1.35 ± 1 a | 1.03 ± 0.86 a | 1.4 ± 1.62 a |
| *Sistotrema* |  | 0.79 ± 1.95 a | 0.62 ± 1.66 a | 1.78 ± 5.23 a | 1.92 ± 7.65 a |
| *Leucosporidium* |  | 0.89 ± 1.74 a | 0.6 ± 1 a | 0.63 ± 0.79 a | 0.96 ± 1.84 a |
| *Eocronartium* |  | 1.17 ± 4.19 a | 1.46 ± 5.17 a | 0.52 ± 1.56 a | 0.4 ± 1.56 a |

**Table S12.** Relative abundance of phyllophere common ASVs at each distance from mine sites. Mean ± SD are shown. Zero-inflated beta mixed regression models were used to compare mean relative abundance of phyllosphere ASVs. Lowercase letters represent significant differences based on Tukey’s test at the 0.05 level. Significant relationships (significant differences in Tukey test at 0.05 level) are highlighted in bold. Common ASVs were identified based on the presence in at least in 90 % of the samples for bacteria and 70 % of the samples for fungi.

| **ASV** | **Taxonomic information** | | | **Mining Stage** | **Distance (km)** | | | |
| --- | --- | --- | --- | --- | --- | --- | --- | --- |
|  | Order | Family | Genus |  | 0 | 0.05 | 0.2 | 1 |
| **Bacterial ASV** | | | | | | | | |
| ASV1 | Burkholderiales | Burkholderiaceae | *Limnobacter* |  | 0.02 ± 0.01 a | 0.02 ± 0.01 a | 0.02 ± 0.01 a | 0.02 ± 0.01 a |
| ASV3 | Sphingomonadales | Sphingomonadaceae | *Sphingomonas* |  | 1.45 ± 1.17 a | 1.84 ± 1.64 a | 1.88 ± 1.23 a | 2.07 ± 1.53 a |
| **ASV4 *** | Burkholderiales | Comamonadaceae | *Leptothrix* | Operating | 1.29 ± 1.02 a | 2.23 ± 1.54 b | 2.2 ± 1.12 b | 2.38 ± 1.64 b |
|  |  |  |  | Non-Operating | 2.13 ± 1.21 a | 2.15 ± 1.03 a | 1.98 ± 1.2 a | 1.96 ± 1.19 a |
| ASV5 | Acetobacterales | Acetobacteraceae | *Acidiphilium* |  | 1.35 ± 1.47 a | 1.36 ± 0.98 a | 1.94 ± 1.72 a | 1.52 ± 1.04 a |
| **ASV6 *** | Burkholderiales | Burkholderiaceae | *Caballeronia* |  | 0.91 ± 1.31 a | 1.13 ± 0.89 ab | 1.4 ± 1.18 b | 1.65 ± 1.17 b |
| ASV7 | Burkholderiales | Comamonadaceae | *Rhizobacter* |  | 1.21 ± 0.81 a | 1.07 ± 0.68 a | 1.32 ± 0.74 a | 0.97 ± 0.66 a |
| ASV8 | Acetobacterales | Acetobacteraceae | *NA* |  | 0.61 ± 0.8 a | 0.88 ± 0.77 a | 1.37 ± 1.44 a | 1.04 ± 0.91 a |
| ASV10 | Acidobacteriales | Acidobacteriaceae | *Terriglobus* |  | 0.67 ± 0.57 a | 0.97 ± 1.59 a | 0.93 ± 0.63 a | 0.97 ± 0.71 a |
| ASV12 | Burkholderiales | Comamonadaceae | *Rhizobacter* |  | 0.85 ± 0.9 a | 0.77 ± 0.58 a | 0.97 ± 0.7 a | 0.72 ± 0.56 a |
| ASV14 | Acidobacteriales | Acidobacteriaceae | *Granulicella* |  | 0.53 ± 0.38 a | 0.76 ± 0.64 a | 0.56 ± 0.33 a | 0.7 ± 0.35 a |
| **ASV15 *** | Sphingomonadales | Sphingomonadaceae | *Sphingomonas* |  | 0.59 ± 0.44 ab | 0.78 ± 0.55 b | 0.6 ± 0.5 ab | 0.48 ± 0.37 a |
| ASV16 | WD260 | NA | *NA* |  | 0.39 ± 0.37 a | 0.39 ± 0.37 a | 0.53 ± 0.46 a | 0.66 ± 0.6 a |
| **ASV17 *** | Sphingomonadales | Sphingomonadaceae | *Sphingomonas* | Operating | 0.51 ± 0.63 a | 0.44 ± 0.33 a | 0.78 ± 0.81 a | 0.46 ± 0.35 a |
|  |  |  |  | Non-Operating | 0.57 ± 0.33 ab | 0.53 ± 0.44 ab | 0.38 ± 0.34 a | 0.71 ± 0.45 b |
| ASV18 | WD260 | NA | *NA* |  | 0.39 ± 0.39 a | 0.37 ± 0.26 a | 0.54 ± 0.42 a | 0.7 ± 0.77 a |
| ASV20 | Acidobacteriales | Acidobacteriaceae | *Granulicella* |  | 0.39 ± 0.37 a | 0.48 ± 0.42 a | 0.41 ± 0.27 a | 0.44 ± 0.32 a |
| **ASV21*** | Acidobacteriales | Acidobacteriaceae | *Granulicella* | Operating | 0.14 ± 0.16 a | 0.21 ± 0.24 a | 0.4 ± 0.44 ab | 0.7 ± 0.69 b |
|  |  |  |  | Non-Operating | 0.49 ± 0.42 a | 0.55 ± 0.43 a | 0.6 ± 0.4 a | 0.54 ± 0.42 a |
| **ASV22*** | Rhizobiales | Beijerinckiaceae | *1174-901-12* |  | 0.41 ± 0.36 a | 0.49 ± 0.39 ab | 0.6 ± 0.47 ab | 0.66 ± 0.44 b |
| ASV23 | Acidobacteriales | Acidobacteriaceae | *Granulicella* |  | 0.46 ± 0.42 a | 0.39 ± 0.27 a | 0.38 ± 0.37 a | 0.4 ± 0.35 a |
| **ASV25*** | Acidobacteriales | Acidobacteriaceae | *Terriglobus* |  | 0.41 ± 0.47 a | 0.44 ± 0.44 a | 0.66 ± 0.71 b | 0.5 ± 0.51 ab |
| **ASV35*** | Acidobacteriales | Acidobacteriaceae | *Granulicella* | Operating | 0.16 ± 0.14 a | 0.3 ± 0.26 ab | 0.57 ± 0.4 c | 0.44 ± 0.29 bc |
|  |  |  |  | Non-Operating | 0.36 ± 0.26 a | 0.36 ± 0.22 a | 0.29 ± 0.28 a | 0.46 ± 0.39 a |
| ASV37 | Acetobacterales | Acetobacteraceae | *NA* |  | 0.31 ± 0.28 a | 0.35 ± 0.42 a | 0.44 ± 0.34 a | 0.39 ± 0.38 a |
| **Fungal ASV** | | | | | | | | |
| ASV2 | Pleosporales | Sporormiaceae | *Sporormiella* |  | 2.15 ± 3.66 a | 3.76 ± 8.04 a | 2.84 ± 6.2 a | 3.53 ± 8.13 a |
| ASV3 | Kriegeriales | Kriegeriaceae | *Phenoliferia* |  | 1.64 ± 1.64 a | 2.27 ± 1.67 a | 2.71 ± 3.03 a | 2.06 ± 2.36 a |
| ASV4 | Chaetothyriales | NA | *NA* |  | 2.38 ± 3.14 a | 1.73 ± 2.5 a | 2.51 ± 2.38 a | 1.95 ± 1.99 a |
| ASV5 | Kriegeriales | Kriegeriaceae | *Phenoliferia* |  | 1.52 ± 2.08 a | 1.39 ± 1.39 a | 1.52 ± 1.28 a | 1.44 ± 1.49 a |
| ASV6 | NA | NA | *NA* |  | 1.24 ± 1.43 a | 1.56 ± 1.94 a | 1.05 ± 1.09 a | 1.13 ± 1.5 a |
| **ASV10 *** | Kriegeriales | Kriegeriaceae | *Phenoliferia* | Operating | 1.57 ± 1.69 b | 1.43 ± 1.12 b | 1.15 ± 0.99 b | 0.5 ± 0.53 a |
|  |  |  |  | Non-Operating | 0.73 ± 1.42 a | 0.66 ± 0.91 a | 0.33 ± 0.35 a | 0.53 ± 0.52 a |
| **ASV13 *** | Spizellomycetales | Powellomycetaceae | *Thoreauomyces* | Operating | 0.37 ± 0.47 ab | 0.42 ± 0.52 a | 1.05 ± 1.02 bc | 1.05 ± 1.07 c |
|  |  |  |  | Non-Operating | 0.67 ± 0.53 a | 1.01 ± 0.8 a | 0.85 ± 0.87 a | 1.04 ± 1.32 a |
| **ASV21*** | Spizellomycetales | NA | *NA* |  | 0.71 ± 0.76 a | 0.65 ± 0.9 a | 0.63 ± 1.13 ab | 0.32 ± 0.74 b |
| **ASV22 *** | Capnodiales | Cladosporiaceae | *Cladosporium* |  | 0.93 ± 1.42 b | 0.61 ± 0.7 ab | 0.37 ± 0.34 ab | 0.4 ± 0.4 a |
| ASV23 | Chaetothyriales | Epibryaceae | *Epibryon* |  | 0.4 ± 0.38 a | 0.63 ± 0.73 a | 0.53 ± 0.51 a | 0.51 ± 0.48 a |
| ASV26 | Chaetothyriales | NA | *NA* |  | 0.39 ± 0.68 a | 0.4 ± 1.06 a | 0.59 ± 1.04 a | 0.58 ± 1.09 a |
| **ASV28 *** | Capnodiales | NA | *NA* |  | 0.42 ± 0.53 a | 0.43 ± 0.49 ab | 0.48 ± 0.43 ab | 0.54 ± 0.52 b |
| ASV32 | Dothideales | Dothioraceae | *Hormonema* |  | 0.39 ± 0.54 a | 0.34 ± 0.34 a | 0.51 ± 0.63 a | 0.51 ± 0.73 a |
| **ASV43 *** | Helotiales | Hyaloscyphaceae | *Polydesmia* |  | 0.24 ± 0.7 a | 0.29 ± 0.36 ab | 0.47 ± 1.12 ab | 0.72 ± 1.06 b |
| ASV54 | Chaetothyriales | Herpotrichiellaceae | *Capronia* |  | 0.29 ± 0.32 a | 0.33 ± 0.46 a | 0.26 ± 0.24 a | 0.44 ± 0.44 a |
| ASV56 | NA | NA | *NA* |  | 0.22 ± 0.28 a | 0.35 ± 0.45 a | 0.43 ± 0.45 a | 0.41 ± 0.59 a |
| **ASV66 *** | Dothideales | Aureobasidiaceae | *Aureobasidium* |  | 0.77 ± 1.47 b | 0.27 ± 0.73 ab | 0.09 ± 0.13 a | 0.11 ± 0.16 a |
| ASV69 | NA | NA | *NA* |  | 0.25 ± 0.43 a | 0.24 ± 0.38 a | 0.26 ± 0.36 a | 0.26 ± 0.39 a |
| ASV95 | Pleosporales | Pleosporaceae | *Alternaria* |  | 0.27 ± 0.5 b | 0.26 ± 0.64 ab | 0.09 ± 0.1 ab | 0.11 ± 0.17 a |
| ASv116 | Orbiliales | Orbiliaceae | *NA* |  | 0.13 ± 0.22 a | 0.19 ± 0.24 a | 0.18 ± 0.2 a | 0.13 ± 0.18 a |


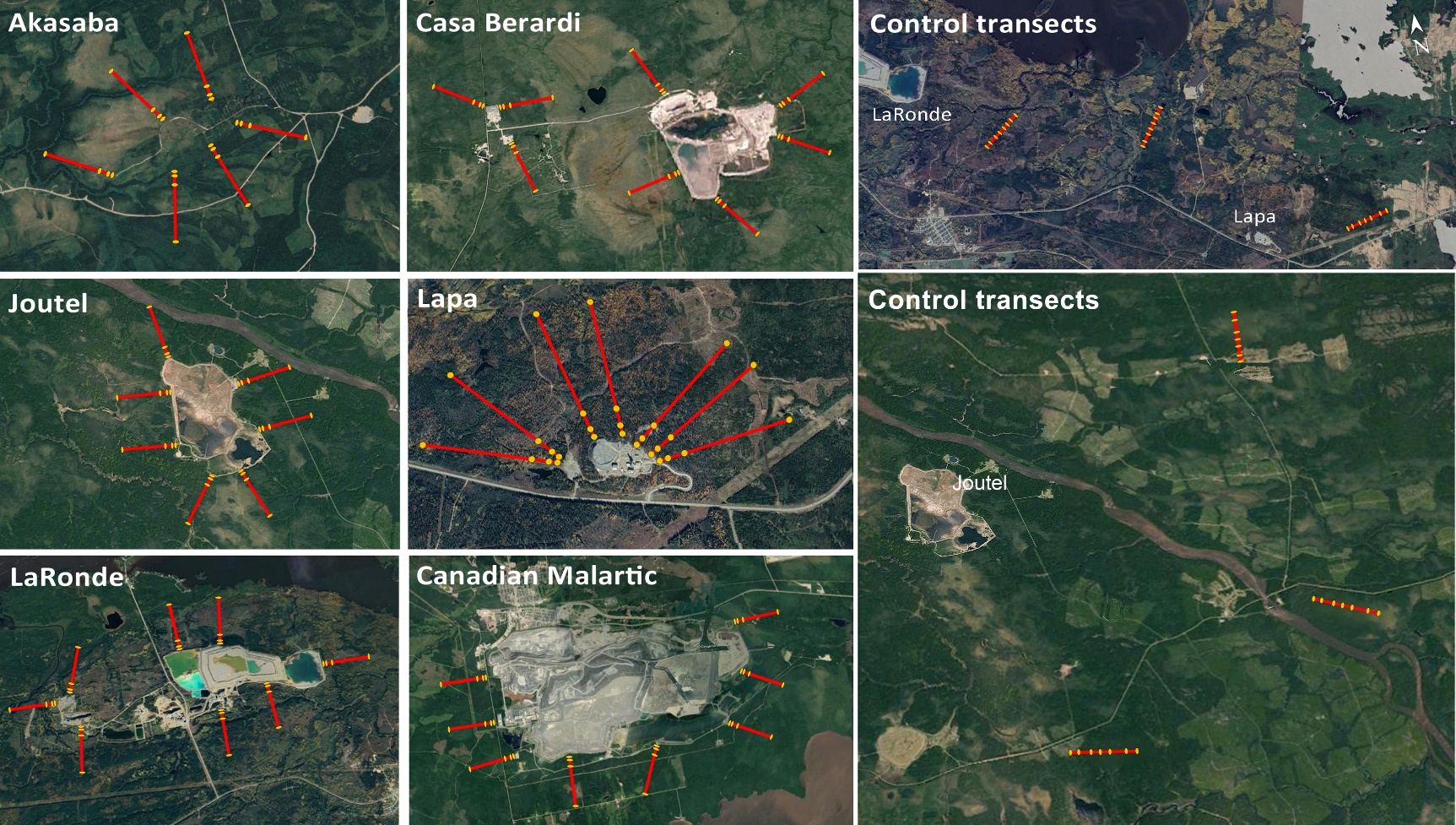


**Fig. S1.** Maps of the study sites and the position of transects. Six selected mine sites and controls: Akasaba site, 6 transects, 24 plots; Casa Berardi site, 8 transects, 32 plots; Joutel site, 7 transects, 28 plots; Lapa site, 7 transects, 28 plots; LaRonde site, 8 transects, 32 plots; Canadian Malartic site, 8 transects, 32 plots; Control transects, 6 transects, 44 plots **.** Scale is not shown because the 1 km transects can be used as a reference in each map.


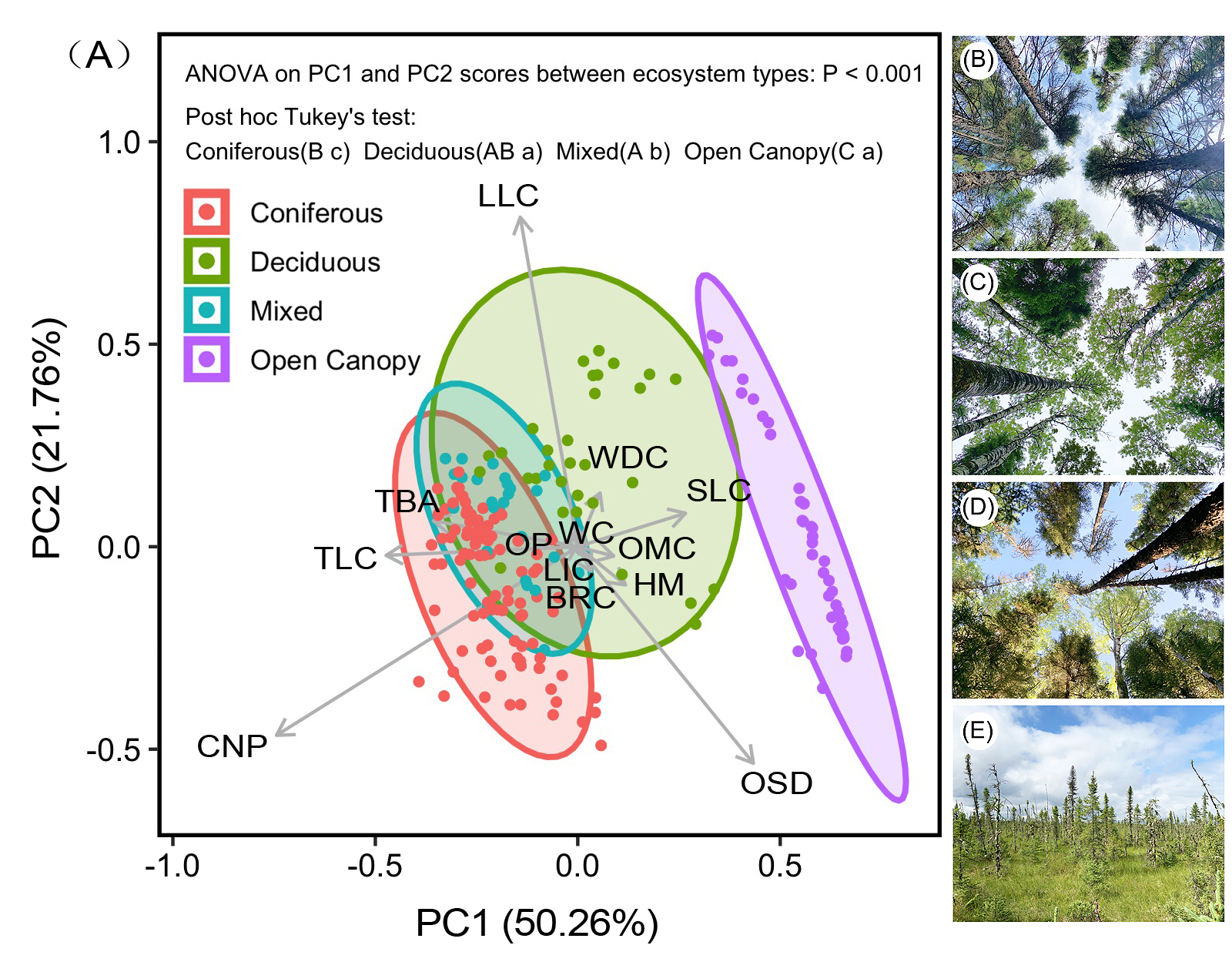


**Fig. S2.** Biplot of principal component analysis (PCA) of forest structures and environmental variables across plots and forest types. Each ecosystem type (coniferous (B), deciduous (C), mixed forest (D) and open canopy ecosystem(E)) is represented by a different color and ellipse, and all plots are displayed (small circles). SLC: live crown ratio for sapling trees; TLC: live crown ratio for canopy trees; SBA: base areas for sapling trees; TBA: base areas for canopy trees; OP: Openness; LLC: leave litter ground coverage; WDC: woody debris ground coverage; WC: water ground coverage; RC: rock ground coverage; OMC: organic matter ground coverage; OSD: depth of organic soil; CNP: conifer proportion; HM: humility; BRC: bryophyte ground coverage; LIC: lichen ground coverage.
